# Evolutionary Reprogramming of Acyltransferase Domains in Polyene Macrolide Pathways

**DOI:** 10.3390/microorganisms14010141

**Published:** 2026-01-08

**Authors:** Liran Zhang, Jinwei Ren, Chengyu Zhang, Lixin Zhang, Bin Wang, Jingyu Zhang

**Affiliations:** 1 State Key Laboratory of Bioreactor Engineering, School of Biotechnology, East China University of Science and Technology, Shanghai 200237, China; y30220602@mail.ecust.edu.cn (L.Z.); ton_nom@163.com (C.Z.); lxzhang@ecust.edu.cn (L.Z.); 2 State Key Laboratory of Microbial Diversity and Innovative Utilization, Institute of Microbiology, Chinese Academy of Sciences, Beijing 100101, China; renjw@im.ac.cn (J.R.); wangbin@im.ac.cn (B.W.); 3 University of Chinese Academy of Sciences, Beijing 100101, China; 4 Beijing Key Laboratory of Genetic Element Biosourcing & Intelligent Design for Biomanufacturing, Beijing 100101, China

**Keywords:** polyketide synthase, assembly line biosynthesis, polyene, acyltransferase, evolution, eurocidin, rimocidin

## Abstract

The evolution of type I polyketide synthase (T1PKS) assembly lines remains poorly understood. Through systematic mining of polyene biosynthetic gene clusters, we identified a novel eurocidin biosynthetic pathway capable of producing identical compounds with divergent loading module architectures, thereby capturing an evolutionary transitional state. Biochemical analysis revealed unprecedented functional reprogramming of acyltransferase (AT) domains, shifting substrate specificity from extender units (malonyl-CoA) to starter units (acyl-CoA). This paradigm shift enables direct initiation of polyketide chain assembly via AT-mediated loading of starter units, thereby elucidating the origin of extant AT-initiated assembly lines and establishing AT functional plasticity as a novel mechanism for polyketide structural diversification. Parallel evolution of ketosynthase (KS) domains through KSS→KSQ mutations further diversified initiation strategies. Applying this evolutionary insight, we engineered the candicidin pathway by replacing its native aromatic-starting bimodule with a starter-selective monomodule from eurocidin, generating aliphatic-starting analogs. This demonstrates that evolution-inspired AT reprogramming provides a rational framework for modifying polyketide starter units, expanding structural diversity, and enhancing therapeutic potential.

## 1. Introduction

Novel biological functions often emerge from the hierarchical organization of molecular components into higher-order machineries. This principle is exemplified by type I polyketide synthases (T1PKSs) and nonribosomal peptide synthetases (NRPSs), where catalytic modules are organized into assembly lines that elongate structures sequentially with exquisite precision. Within a canonical T1PKS extension module, an acyltransferase (AT) domain loads an extender unit (e.g., malonyl-CoA) to an acyl carrier protein (ACP) domain, then a ketosynthase (KS) domain catalyzes decarboxylative Claisen condensation with the upstream acyl chain, and finally coordinated β-carbon processing is carried out by auxiliary domains such as ketoreductase (KR), dehydratase (DH), enoylreductase (ER), and methyltransferase (MT), before chain transfer to the next module. The composition and sequential order of these modules dictate the framework of the final metabolites, underpinning the predictive power of this biosynthetic logic [1,2].

**Figure 1:**
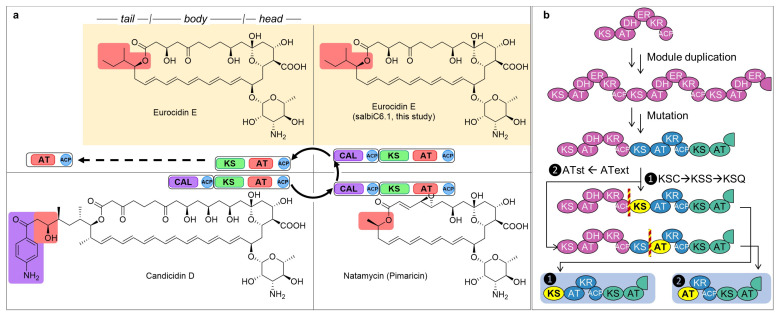
Representative polyene macrolides and hypothesis for assembly line T1PKS evolution. (**a**) Chemical structures are colored, acyl (red) and aromatic (purple), matching the colors of their cognate loading module domains. Brown shading highlights the eurocidin systems undergoing an evolutionary transition. (**b**) Proposed parallel pathways based on KS specialization toward KSQ and AT transition from extender-unit (AText) to starter-unit (ATst) selectivity, explaining the origin of KS- and AT-starting assembly lines.

Polyene macrolides, a class of T1PKS products with exceptional antifungal activity [3,4], represent a compelling system to investigate the evolutionary plasticity of the assembly line biosynthesis (Figure S1) [5]. They feature a tripartite structure: a mycosamine-modified hydrophilic head (mediating ergosterol binding), a polyene-polyol body (for membrane insertion or oligomerization), and a hydrophobic tail (Figure 1a). Arising from differential T1PKS initiation, the tails diverge from simple aliphatic acyl groups to aromatic moieties. Significantly, this initiation itself reveals an architectural anomaly.

To elaborate, the biosynthesis of the heptaene macrolide candicidin [6], which is initiated by an aromatic group, employs a bimodular architecture: CAL(CoA-ligase)-ACP-KS-AT-ACP. Within this system, the CAL domain activates the *p*-aminobenzoate unit, the AT domain recognizes the extender unit malonyl-CoA, and the KS domain catalyzes the decarboxylation of the extender unit, followed by a Claisen condensation with the *p*-aminobenzoate unit loaded onto the upstream ACP. Intriguingly, this same bimodular organization is also present in other aliphatic-starting pathways, such as natamycin [7]. However, in these pathways, the CAL domain is functionally inactive, and the catalytic site of the KS domain undergoes a specific mutation—from Cys to Ser, resulting in the KSS subtype. This altered KS domain only performs decarboxylation of the extender unit delivered by the AT domain, thereby facilitating polyketide chain elongation [7]. Functionally, therefore, these systems closely resemble the common monomodular initiation forms: either KSS-AT-ACP or KSQ-AT-ACP. While the KSS-AT-ACP configuration is prevalent in the BGCs of aliphatic-starting polyenes such as nystatin [8] and amphotericin B [9], the KSQ-AT-ACP architecture—featuring a Gln residue in place of the active-site Cys—is typically associated with non-polyene polyketides like meridamycin [10].

In other words, these bimodular systems have evolved toward functional monomodularity while structurally retaining the bimodular features characteristic of aromatic-starting pathways. This decoupling of structure and function suggests that the system is undergoing an evolutionary transition, offering a valuable opportunity to elucidate the mechanisms underlying the gradual loss of loading modules during evolution.

Therefore, this study was designed to capture such “evolutionary snapshots” in polyene T1PKS initiation systems. In particular, we seek BGC pairs that direct biosynthesis of identical metabolites but differ specifically in their loading mono- or bimodular architectures. These cases would provide direct evidence for the evolutionary trajectory from bimodular to monomodular initiation and allow elucidation of the underlying structural and mechanistic determinants governing this transition. Building on this evolutionary framework, we further explore its utility for rational engineering of novel candicidin analogs.

## 2. Materials and Methods

### 2.1. Bacterial Strains, Culture Conditions, and General Remarks

*Escherichia coli* strains were cultured in Luria-Bertani broth (LB) at 37 °C supplemented with 50 μg/mL of kanamycin when needed. *E. coli* DH5α was used for cloning and BL21(DE3) for induced protein expression. *E. coli* ET12567(pUZ8002) was used for conjugation. *Streptomyces albireticuli* NRRL B-1670 was cultured on ISP4 solid medium at 30 °C for 4–5 days before spores were harvested. *S. albus* J1074, the candicidin-producing strain, was cultured on MS medium for 4–5 days to collect fresh spores. DNA polymerase was purchased from Vazyme, Nanjing, China, restriction enzymes from New England Biolabs (NEB), Beijing, China, and competent cells from Tsingke, Beijing, China. Malonyl-CoA, methylmalonyl-CoA, ethylmalonyl-CoA, acetyl-CoA, propionyl-CoA, butyryl-CoA, and isobutyryl-CoA were purchased from Macklin, Shanghai, China. Acetyl-SNAC, propionyl-SNAC, and isobutyryl-SNAC were chemically synthesized.

### 2.2. Plasmid Construction, Protein Expression and Purification

Plasmids were constructed using the Gibson Assembly method. Target gene fragments were amplified by PCR using 2× Phanta Flash Master Mix and subsequently assembled into the predigested N-His tag backbone pET28a/HindIII + BamHI expression vector with Assembly Mix Ultra. All constructs were verified by DNA sequencing. Correct plasmids were transformed into *E. coli* BL21(DE3) and cultured in LB liquid medium containing 50 μg/mL kanamycin. When OD_600_ reached 0.4–0.6, protein expression was induced with 0.1 mM isopropyl β-D-1-thiogalactopyranoside (IPTG), at 18 °C, 180 rpm, overnight. Protein purification followed the protocol of Ni-NTA his-tag affinity chromatography. The purified protein was collected, concentrated by ultrafiltration, diluted with an equal volume of 80% glycerol, and stored at −20 °C. The AT and primer sequences used in this study are listed in Table S1 and Table S2, respectively.

### 2.3. Fermentation Optimization and Product Analysis of S. albireticuli

Fresh spores of *S. albireticuli* were inoculated into three liquid media with different carbon sources—MS, MYM, and ISP4—and cultured at 28 °C, 250 rpm for 4 days. The fermentation broths were then collected, concentrated by rotary evaporation, and analyzed by high-performance liquid chromatography (HPLC) using a Shim-packGISTC18 (4.6 × 250 mm, 5 mm). The HPLC analysis was performed with solvent A: water with 0.1% TFA and B: acetonitrile with 0.1% TFA. The gradient program was set as follows: 0–15 min, 5% to 50% B; 15–20 min, 50% to 100% B; 20–25 min, 100% B; 25–26 min, 100% to 5% B; 26–30 min, 5% B. The detection wavelength was set at 350 nm, and the flow rate was 1 mL/min. The extraction and HPLC quantification of fermentation products were based on three independent fermentation experiments, with each fermentation sample analyzed in duplicate by HPLC. MS medium yielded a higher quantity of target compounds. Therefore, MS was selected as the fermentation medium for subsequent scale-up fermentation.

### 2.4. Fermentation and Purification of S. albireticuli Metabolites

A seed culture was prepared by inoculating fresh spores of Streptomyces albireticuli into 100 mL of MS medium (28 °C, 250 rpm, 1–2 days). This seed culture was then used to inoculate 200 mL of the same medium in shake flasks at a 5% (*v*/*v*) inoculum size, with a total fermentation volume of 4 L. After growing for 4–5 days with 2% (*w*/*v*) XAD-16 resin, the biomass and resin were collected and extracted three times with methanol. The combined extracts were concentrated by rotary evaporation and isolated by a packed C18 resin (6 cm × 20 cm) with a 10–100% methanol-water gradient. The column fractions were collected and analyzed by HPLC on a Shim-packGISTC18 column to detect the target product. Fractions containing the target compound were pooled and further purified by semi-preparative HPLC using a GH0525010C18AQ column (10 mm × 250 mm, 5 mm), using water (A) and acetonitrile (B). The HPLC method: 0–15 min, 10% to 26% B; 15–19 min, 26% B; 19–20 min, 26% to 100% B; 20–24 min, 100% B; 24–25 min, 100% to 10% B, with a flow rate of 3 mL/min and detection at 350 nm. After overnight freeze-drying, After overnight freeze-drying, 4 mg of eurocidin E, 3.5 mg of eurocidin E1, and 3.0 mg of eurocidin E2 were obtained. To obtain a sufficient amount of eurocidin E2 for subsequent structural characterization, an additional 4 L fermentation was subsequently carried out under the same conditions. The purified compounds were dissolved in 500 μL of DMSO-*d6*, and characterized by nuclear magnetic resonance (NMR). All spectra were recorded on a Bruker Avance III 500 MHz spectrometer.

### 2.5. In Vitro Enzymatic Assay

The AT activity was measured in a reaction mixture containing 50 mM Tris-HCl (pH 8.0), 20 mM AT (and mutants), and 1 mM of extender unit substrates (malonyl-CoA, methylmalonyl-CoA and ethylmalony-CoA) or starter unit substrates (acetyl-CoA, propionyl-CoA, butyryl-CoA, isobutyryl-CoA and isovaleryl CoA). The reaction was incubated at 30 °C for 1 h and subsequently terminated by adding equal volume of methanol. All experiments were performed in three independent replicates. The production of CoA was detected by HPLC using a ShimNex WP C18-S column (4.6 mm × 100 mm, 5 mm). Solvent A was water with 0.1% trifluoroacetic acid (TFA), and Solvent B was acetonitrile with 0.1% TFA. The elution was carried out at a flow rate of 1.0 mL/min with gradient program: 0–6 min, 5% to 30% B; 6–7 min, 30% to 100% B; 7–9 min, 100% B; from, 9–10 min, 100% to 5% B; 10–12.5 min, 5% B. The detection wavelength was set at 256 nm.

### 2.6. Construction of the S. albus J1074 ΔfscA

The *fscA* gene encodes the loading module of candicidin. The *S. albus J1074 ΔfscA* mutant was generated by double-crossover homologous recombination. The 1.5 kb upstream and downstream flanking regions of the *fscA* gene were PCR-amplified and cloned into the temperature-sensitive vector pKC1139, creating the knockout plasmid pKC1139-*fscA*. The resulting plasmid was transformed into *E. coli* ET12567(pUZ8002) before conjugation into *S. albus* J1074. The *fscA* knockout mutant was finally confirmed by PCR and metabolic profiling by HPLC.

### 2.7. Precursor-Directed Feeding Assay in S. albus J1074 ΔfscA

The substrate analogs acyl-N-acetylcysteamines (acyl-SNACs) need to be synthesized. SNAC (1 mmol) and carboxylic acid (1 mmol, acetic acid or propionic acid) were dissolved in 4 mL of dichloromethane with stirring at room temperature. DMAP (0.1 mmol, 12.3 mg) and EDC (1.1 mmol, 216 mg) were then added sequentially. The mixture was stirred at room temperature for 30 min and monitored by thin-layer chromatography (TLC) or HPLC. After the reaction was complete, the crude product was adsorbed onto silica gel, dried, and purified by column chromatography (silica gel, 15 cm × 3 cm) using petroleum ether/ethyl acetate (1:5, *v*/*v*) as the eluent. The eluate was collected as 5 mL fractions, and the composition of each fraction was monitored by TLC. Fractions containing the target product were pooled, concentrated by rotary evaporation to remove the solvent, and then redissolved in a suitable volume of DMSO for storage. For the feeding experiment, acetyl-SNAC or propionyl-SNAC was added to 50 mL of sterilized MS liquid medium to achieve a final concentration of 1 mM. Fresh spores of the *ΔfscA* mutant were then inoculated and cultured at 28 °C and 250 rpm for 4–5 days. Following fermentation, the broth was processed and analyzed by HPLC as described in Section 2.3.

### 2.8. Heterologous Module Complementation in S. albus J1074 ΔfscA

The salbiC6.1 loading didomain and the “salbiC6.1 loading domain (LD) + *fscA* docking domain (DD)” were respectively cloned into the pSET152 vector containing the kasOp* promoter, and the correctness of plasmid construction was confirmed by DNA sequencing. The two constructed plasmids were individually transformed into *E. coli* ET12567(pUZ8002) and then introduced into the *ΔfscA* mutant via conjugation. Recombinant strains were finally verified by PCR, yielding the correctly constructed strains *ΔfscA-salbiC6.1 LD* and *ΔfscA-salbiC6.1LD + CandDD*. These two recombinant strains were streaked densely onto MS solid medium and incubated upside-down at 30 °C for 4–5 days, with the *ΔfscA* mutant and wild-type *S. albus* J1074 cultured in parallel as controls. After fermentation, the solid medium was cut into small pieces and soaked in methanol for approximately 6 h to extract metabolites. The methanol extracts were collected, concentrated by rotary evaporation, and then analyzed by HPLC to compare product profiles.

## 3. Results

### 3.1. Evolution of the Loading Module from Bimodular to Monomodular in Eurocidin biosynthesis

**Figure 2:**
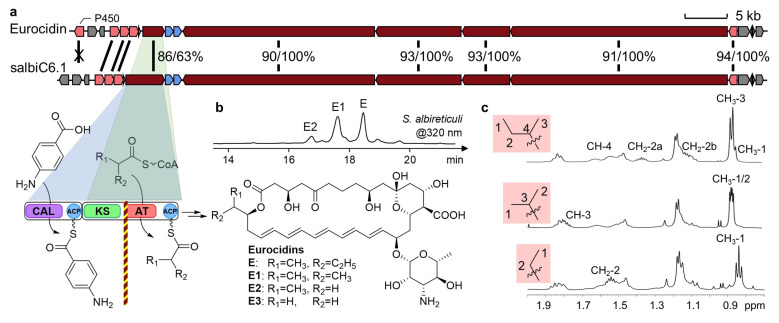
Characterization of salbiC6.1 as a new eurocidin BGC. (**a**) Comparative genomics of eurocidin BGCs, showing gene coverage/identity% for T1PKS genes and highlighting divergent loading module architectures. A vertical barrier denotes functional insulation from upstream domains. (**b**) HPLC analysis of polyene products from *S. albireticuli* cultivation. (**c**) ^1^H NMR spectra demonstrating the distinct acyl groups of isolated eurocidin congeners (from top to bottom: E, E1 and E2).

To pursue the objective mentioned in the Instruction section, we mined the antiSMASH database [11,12] for homologs of reference polyene BGCs (Figure S1), focusing on candidates with conserved overall architecture yet divergent loading modules. However, only one tetramycin-like and two eurocidin-like BGCs were obtained. Subsequent inspection confirmed the tetramycin-like BGC to be identical to the reference, while the eurocidin-like BGCs display architectural variations unrelated to the loading module (Figure S2). Using the highly conserved hexamodular AmphI [13] sequence from the amphotericin BGC, which is responsible for synthesizing the polyene-hemiketal domain, we performed a tblastn search against the NCBI non-redundant (nr) database and identified a eurocidin-like biosynthetic gene cluster from *S. albireticuli* NRRL B-1670 (salbiC6.1). This cluster was the only candidate that met the screening criteria (Figure 2). Cultivation of this strain yielded compounds with characteristic polyene UV-vis absorption, from which three pentaene products were isolated and characterized by NMR as eurocidins E [14,15], E1, and E2 (Figure 2 and Figures S3–S23). LC-HRMS analysis identified a fourth congener, E3, containing an acetyl starter unit; notably, no (amino)benzoate-derived analogs could be detected (Figure S4), indicating a nonfunctional CAL-ACP module. Although targeted deletion of the CAL-ACP region to confirm dispensability was unsuccessful, the production of aliphatic-starting eurocidins by these architecturally divergent loading modules provides direct evidence for an evolutionary trajectory from bimodular to monomodular initiation.

### 3.2. Functional Reprogramming of Polyene Polyketide Synthase AT Domains: Evolution from Extender Unit to Starter Unit Selection

**Figure 3:**
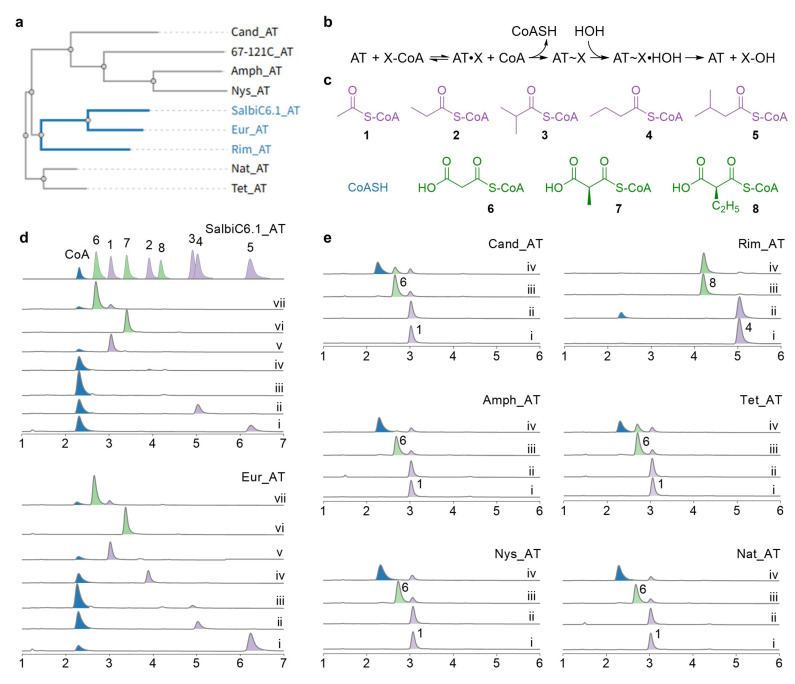
Enzymatic analysis of AT domains from polyene loading modules. (**a**) Phylogenetic analysis reveals a subclade within the polyene clade (Figure S24) comprising salbiC6.1_AT, Eur_AT, and Rim_AT that selectively recognize acyl-CoA starter units, separate from malonyl-CoA-selecting AT domains. (**b**) Scheme of the in vitro AT activity assay. (**c**) Chemical structures of acyl-CoA substrates used in this study. (**d**) Activity profiles of SalbiC6.1_AT and Eur_AT showing specific hydrolysis of acyl-CoA substrates (traces i–v, 5–1) but not malonyl-CoA derivatives (traces vi–vii, 7–6). The top curve displays all CoA derivative standards. (**e**) Substrate specificity of additional polyene AT domains showing that Rim_AT activates acyl-CoA while other ATs recognize malonyl-CoA. Control reactions (traces i and iii) used heat-inactivated enzyme. The HPLC peaks in (**d**,**e**) are labeled in green (extender units), light purple (starter units), and dark blue (CoA), corresponding to the color scheme used in (**c**).

The structural profiles of the isolated eurocidin congeners suggest direct selection from acyl-CoA starter pools, contradicting the phylogenetic analysis that places all polyene loading module AT domains within the extender unit-selecting clade (Figure S24 and Figure 3a) and recent characterization of the natamycin AT domain [7]. This discrepancy prompted experimental validation. We conducted in vitro analysis [16] of purified AT domains from both eurocidin BGCs reacting with a set of CoA derivatives (Figure 3d and Figure S25), which demonstrated specific hydrolysis of short-chain acyl-CoAs but no activity toward malonyl- or methylmalonyl-CoA (Figure 3d). This substrate profile aligned precisely with the congener distribution, thus firmly establishing their starter unit selection function.

To explore the functional implications of the phylogenetic clustering, we purified and assayed AT domains from all characterized polyene BGCs [7,9,17,18,19] (Figure 3e and Figure S25 and Table S1). Notably, the rimocidin system presented an unexpected biosynthetic logic: while it employs a crotonyl-CoA carboxylase/reductase (CCR) [20,21] for ethylmalonyl-CoA biosynthesis for the last extension module, its loading AT domain directly activates butyryl-CoA. In contrast, all other polyene loading AT domains demonstrated strict malonyl-CoA specificity, consistent with their respective product profiles.

The close phylogenetic relationship between the eurocidin and rimocidin AT domains suggests that they might be recently derived starter AT variants, thus remaining distinct from canonical ones (Figure 3a). This mechanistic reprogramming from extender-unit to starter-unit selection represents a fundamental evolutionary innovation by establishing an initiation mechanism independent of KS-mediated decarboxylation. This new capability incidentally renders upstream module/domains functionally redundant, in turn, generating selective pressure for their loss, as their metabolic costs provide no compensatory advantage. This pressure is particularly evident in aliphatic-starting pathways, where the dedicated *p*-aminobenzoate biosynthetic genes (including aminodeoxychorismate synthase [6]) are absent, rendering the CAL-ACP module dispensable. Within the established “module duplication” model of T1PKS evolution [22,23,24], this AT transition provides crucial mechanistic evidence explaining the origin of AT-starting assembly lines (Figure 1b), despite not having discovered an extant AT-starting eurocidin BGC.

**Figure A1:**
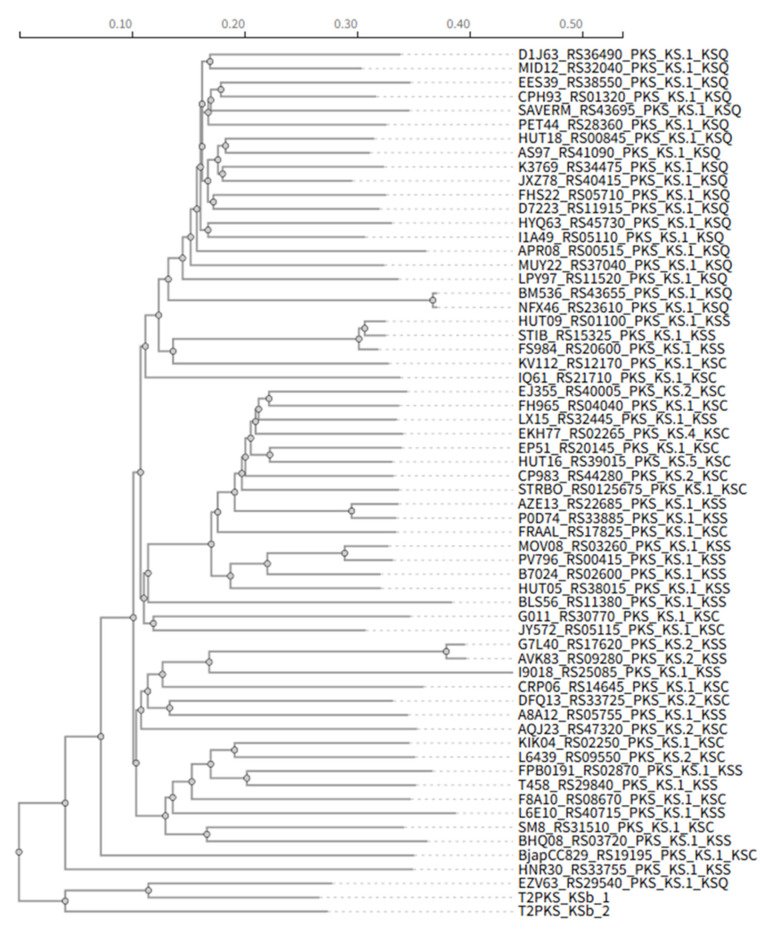
Phylogenetic analysis of KS domains from T1PKS BGCs. Sequences are collected from a local KS database containing KSC, KSS, and KSQ subtypes. For better inspection, 20 sequences from each subtype were randomly picked. With addition of 2 KS domains from T2PKS BGCs from Streptomyces coelicolor, all sequences were subjected to MUSCLE.

Parallel to AT evolution, phylogenetic analysis [25] reveals a distinct KS specialization pathway where domains initially diverge into KSS (Cys->Ser mutation) subtypes that retain decarboxylation activity compatible with extender unit-selecting AT partners while exhibiting reduced Claisen condensation activity [26]. This trajectory culminates in KSQ (Cys->Gln mutation) subtypes that exclusively perform extender unit decarboxylation while completely losing Claisen condensation activity [27,28], thereby insulating upstream domains and ultimately facilitating their evolutionary loss. The predominance of KSQ subtypes in extant KS-initiated assembly lines represents successful evolutionary endpoints of this specialization process (Figure 1b and Figure A1). Together, these complementary evolutionary pathways, AT reprogramming and KS specialization, demonstrate the remarkable plasticity of T1PKS assembly lines [29].

### 3.3. Rational Engineering of Candicidin

Informed by this evolutionary insight into AT domain reprogramming, we next explored its potential for rational engineering of the candicidin pathway because its *p*-aminobenzoyl starter unit, installed by a CAL-dependent bimodular system, has been implicated in its narrow antifungal spectrum and associated toxicity [6]. We hypothesized that replacing this aromatic starter unit with an aliphatic acyl group, leveraging the repurposed AT mechanism we identified, could serve as a strategy to improve its therapeutic properties.

**Figure A2:**
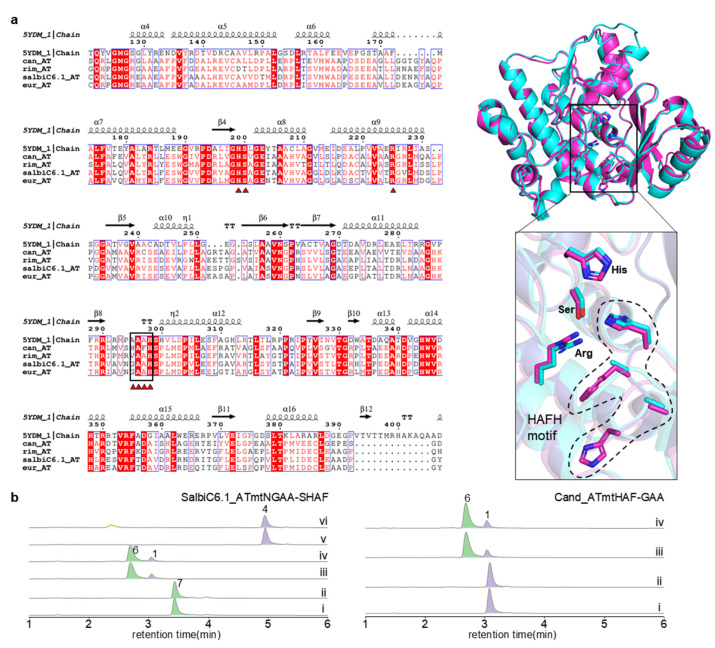
Sequence and structural analyses of AT domains and HPLC analysis of motif swapping mutants. Sequence alignment was performed by ENDscript server and 3D structures were modeled by AlphaFold3. (**a**) Sequence and structure alignment between Salbi_AT and Cand_AT. Triangles correspond to the AT’s critical active sites, and black border to the motifs involved in substrate binding. (**b**) The motif-swapping mutants exhibited an almost complete loss of activity toward the natural substrate while failing to acquire the expected noncognate activity. Control reactions (traces i, iii and v) used heat-inactivated enzyme.

To this end, we first sought to elucidate the molecular basis of the AT domain transition from extender unit to starter unit selectivity. In most cases, AT domains that recognize malonyl-CoA contain a HAFH substrate-binding motif, whereas the loading module AT domain of eurocidin, which recognizes acyl-CoA, possesses a corresponding GAAH binding motif. While sequence alignment [30] revealed 44% identity between eurocidin and candicidin loading AT domains, comparative structural analysis [31] failed to identify clear determinants beyond the conserved HAFH/GAAH motif [32,33,34] (Figure A2a). Motif-swapping [35,36,37] experiments generated chimeric mutants that showed dramatically reduced activities toward cognate CoA substrates while failing to acquire the expected noncognate activity (Figure A2b), agreeing with that CoA substrate discrimination involves residues beyond the immediate active site [34].

**Figure 4:**
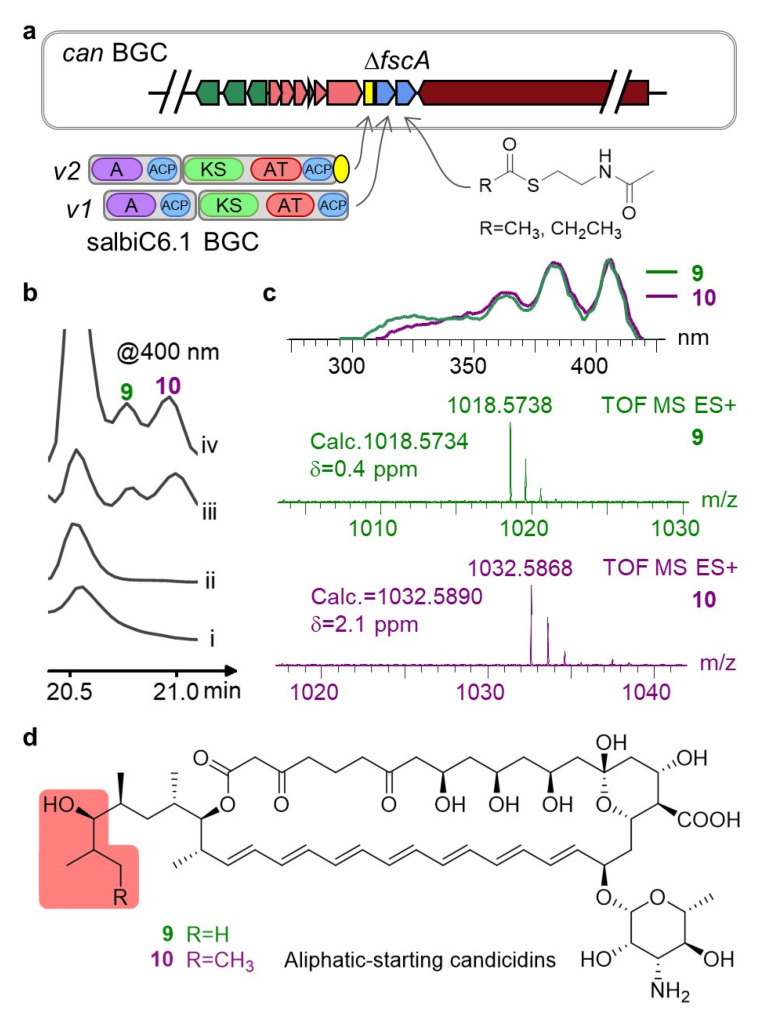
Engineering of the candicidin loading module. (**a**) Schematic of loading bimodular gene *fscA* knockout and complementation with that from salbiC6.1 BGC. The yellow box in can BGC indicates the remnant *fscA* and the yellow oval denotes the C-terminal docking domain from *fscA* fused to the salbiC6.1 loading module. Simple acyl-SNAC were also fed to the knockout strain. (**b**,**c**) HPLC, UV-vis, and HRMS analyses of metabolites from the recombinant strains. Traces in (**b**) denotes (i) wild-type, (ii) knockout strain, (iii,iv) complemented with v1 and v2 from (**a**). (**d**) Chemical structures of aliphatic-starting candicidin analogs.

**Figure A3:**
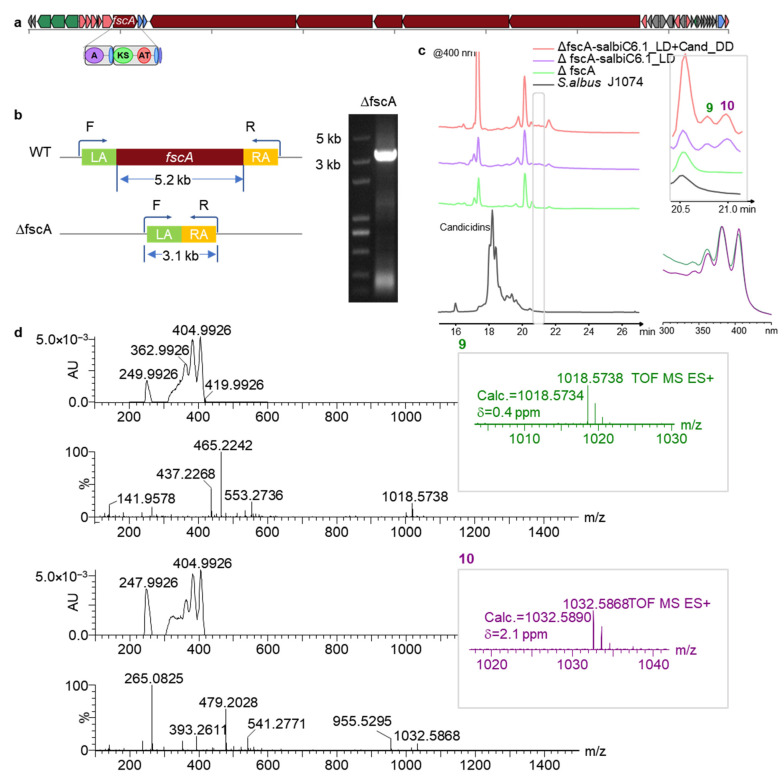
Metabolic profiling of loading module recombinants. (**a**) Composition of the candicidin biosynthetic gene cluster. (**b**) schematic of homologous double-crossover based knockout of *fscA*. (**c**,**d**) HPLC and HRMS analyses of polyene compounds.

We constructed the *ΔfscA* knockout mutant to eliminate the native candicidin loading module and employed precursor-directed biosynthesis by feeding chemically synthesized acyl-SNAC mimics. Despite complete consumption of the precursors, no polyene production was detected, suggesting that the free precursors were not efficiently recognized or processed by the downstream modules. To verify the functional portability of the heterologous loading module, we complemented the *ΔfscA* mutant with the native salbiC6.1 loading module, as well as a modified version fused with the *fscA* docking domain [38] (Figure 4a). Both complemented strains produced compounds displaying characteristic polyene UV absorption, and HRMS confirmed molecular weights corresponding to aliphatic-starting candicidin analogs (Figure 4b–d and Figure A3). Notably, yields were significantly lower than those of the wild-type strain, although the version fused with the docking domain showed a slight improvement in production. These findings confirm the feasibility of replacing the starter unit via AT reprogramming while highlighting that compatibility between heterologous modules and native elongation modules is a critical bottleneck limiting biosynthetic efficiency. Future efforts should focus on optimizing docking interfaces or synergistic folding to enhance pathway flux.

## 4. Discussion

The substrate selectivity mechanism of ATs is not entirely determined by single motifs such as GAAH or HAFH, and the complex structure-activity relationships underlying this mechanism remain to be elucidated. The negative result from our “motif-swapping” strategy, together with several recent studies, reveals a more profound insight: the substrate selectivity of AT is an “emergent property” co-determined by a distributed network of residues, far beyond the control of a single motif.

Simulations of EryAT6 by Kalkreuter et al. [34] showed that residues distal to the YASH motif (specifically in the LSM and SSM) synergistically regulate active site conformation. Structural analysis of the promiscuous SpnD-AT further supports this: its hydrophobic cavity, shaped by residues such as F145 and A268/A270, determines the substrate size limit [39]. In contrast, the equivalent positions in stringent ATs like EryAT6 are occupied by bulky residues (Y278/S280). Therefore, engineering EryAT6 requires replacing these “sterically restricting” residues with smaller ones to expand the cavity, effectively reshaping the global active site topology rather than simply swapping local motifs [39]. Research on salinomycin ATs also supports this view: in addition to the core motif, multiple hydrophobic residues (e.g., I149, F210, V220 in SalAT14) that interact with the α-substituent of the substrate collectively constitute the determinants of selectivity; mutating the motif alone leads to loss of activity, whereas coordinated engineering of these peripheral residues enables efficient switching of substrate specificity [40].

In summary, the reprogramming of AT selectivity from extender units to starter units may involve complex multi-site co-evolution. This process would require not only altering residues that directly contact the substrate but also coordinately adjusting key regions that maintain the overall architecture of the active site, inter-subunit interactions, and channel dynamics to accommodate the unique binding requirements of starter units (e.g., the lack of a carboxylate group and different demands on electrostatic environment, hydrophobicity, and spatial geometry). Future research should shift focus toward systematically identifying all key residues constituting this complex substrate-binding network, integrating deep mutational scanning, AI-based structural prediction (e.g., AlphaFold3), and extensive molecular dynamics simulations of AT-substrate complexes. This integrated approach will elucidate the global structure-activity relationships governing selectivity between “extender” and “starter” units, ultimately enabling the rational design and precise reprogramming of PKS initiation mechanisms.

The low yield of aliphatic-starting candicidin analogs primarily results from the cumulative effects of multiple mechanistic bottlenecks. First, insufficient inter-module docking efficiency is a key limiting factor: the chimeric docking interface used in the study (heterologous salbiC6.1 loading module + native *fscA* docking domain) may not achieve optimal affinity and spatial complementarity, and such non-native combinations can reduce substrate-channeling efficiency [41]. Second, mismatched secondary interactions between catalytic domains further constrain efficiency: beyond the initial binding mediated by docking domains, the fine-tuned interactions between catalytic domains such as ACP and KS are critical for accurate substrate positioning [42], and the introduction of heterologous modules may disrupt these evolutionarily formed interaction networks. Third, kinetic incompatibility exists: the heterologous AT domain may exhibit mismatched catalytic rates and substrate affinity relative to downstream modules within the candicidin assembly line, while heterologous protein expression imposes an additional metabolic burden. Finally, this highlights the inherent limitations of the “unit-replacement” strategy in PKS engineering—successful pathway reconstruction requires systematic optimization of the entire module interface rather than simply swapping functional domains. Future improvements may employ a combination of strategies, including orthogonal synthetic docking tools (e.g., SYNZIPs) [43], directed evolution of interfaces, and kinetic balancing, to systematically address the low-yield issue and achieve high-titer production of novel polyketide analogs.

## 5. Conclusions

In this study, we successfully captured evolutionary transitions in the polyene T1PKS initiation by identifying BGC pairs producing identical compounds through architecturally divergent loading modules. We demonstrated that AT domain reprogramming from extender-unit to starter-unit selectivity represents a key evolutionary innovation. Concurrently, KS domain specialization via Cys→Ser→Gln mutations progressively eliminated Claisen condensation activity while preserving decarboxylation, offering an alternative evolutionary route for assembly line optimization. These two complementary mechanisms—AT functional reprogramming and KS domain specialization—jointly reveal the remarkable plasticity exhibited by T1PKS assembly lines during evolution.

Guided by this evolutionary insight, we rationally engineered the candicidin pathway by replacing its native aromatic-starting bimodule with a starter-selective monomodule from the eurocidin pathway, yielding aliphatic-starting analogs. This demonstrates the feasibility of evolution-inspired engineering to modify polyketide structures, establishing a framework for diversifying starter units, mitigating aromatic-associated toxicity, and enhancing therapeutic potential.

This research not only deepens the understanding of the evolutionary mechanisms of T1PKS assembly lines but also provides new theoretical foundations and technical pathways for the rational design and engineering of polyketide natural products.

## Data Availability

The data presented in this study are available in article and Supplementary Material.

## Supplementary Materials

The following supporting information can be downloaded at: https://www.mdpi.com/article/10.3390/microorganisms14010141/s1, Table S1: AT sequences analyzed in this study; Table S2: Plasmids, primers, and sequences used in this study; Figure S1: Representative polyene macrolides and corresponding biosynthetic loading module architectures; Figure S2: Tetramycin- and eurocidin-like biosynthetic gene clusters identified from antiSMASH database; Figure S3: Metabolic profiles of Streptomyces albireticuli in three media; Figure S4: LC-HRMS data of fermentation products from *S. albireticuli*; Figures S5–S23: NMR spectra for Eurocidin E, E1 and E2; Figure S24: Phylogenetic analysis of AT domains from representative T1PKS BGCs; Figure S25: SDS-PAGE analysis of wild-type and mutant AT domains from loading modules of polyene T1PKS.

## Abbreviations

The following abbreviations are used in this manuscript:

T1PKS	type I polyketide synthase
AT	acyltransferase
HPLC	high-performance liquid chromatography
LC-HRMS	Liquid chromatography-high resolution mass spectrometry
