# Supplementary material for: Evolutionary Reprogramming of Acyltransferase Domains in Polyene Macrolide Pathways
Source: Microorganisms. 2026 Jan 8;14(1):141. doi: 10.3390/microorganisms14010141 (PMC12843615; doi:10.3390/microorganisms14010141)
Supplement: Supplementary file 1 [file microorganisms-14-00141-s001.zip › microorganisms-4032248-supplementary.pdf]

Supplementary Information for

Evolutionary reprogramming of acyltransferase domains in polyene macrolide pathways

Liran Zhang[1], Jinwei Ren[2], Chengyu Zhang[1], Lixin Zhang[1], Bin Wang[2,3,4], and Jingyu Zhang[1,*]

1 State Key Laboratory of Bioreactor Engineering, and School of Biotechnology, East China University of Science and Technology, Shanghai, 200237, China; y30220602@mail.ecust.edu.cn (L.Z.); ton_nom@163.com (C.Z.); lxzhang@ecust.edu.cn (L.Z.); zhangjingyu@ecust.edu.cn (J.Z.)

2 State Key Laboratory of Microbial Diversity and Innovative Utilization, Institute of Microbiology, Chinese Academy of Sciences, Beijing 100101, China; renjw@im.ac.cn (J.R.); wangbin@im.ac.cn (B.W.)

3 University of Chinese Academy of Sciences, Beijing, 100101, China

4 Beijing Key Laboratory of Genetic Element Biosourcing & Intelligent Design for Biomanufacturing, Beijing 100101, China

* Correspondence: zhangjingyu@ecust.edu.cn (J.Z.)

Contents

Table S1. AT sequences analyzed in this study 4

Table S2. Plasmids, primers, and sequences used in this study 8

Supplementary Figures 10

Figure S1. Representative polyene macrolides and corresponding biosynthetic loading module architectures. 10

Figure S2. Tetramycin- and eurocidin-like biosynthetic gene clusters identified from antiSMASH database. 11

Figure S3. Metabolic profiles of *Streptomyces albireticuli* in three media. 12

Figure S4. LC-HRMS data of fermentation products from *S. albireticuli*. 13

Figure S5. ^{1}H-NMR spectrum of eurocidin E in DMSO-d_6. 14

Figure S6. ^{13}C-NMR Spectrum of eurocidin E in DMSO-d_6 15

Figure S7. ^{1}H-^{1}H COSY spectrum of eurocidin E in DMSO-d_6 16

Figure S8. HSQC spectrum of eurocidin E in DMSO-d_6 17

Figure S9. HMBC spectrum of eurocidin E in DMSO-d_6 18

Figure S10. ROESY spectrum of eurocidin E in DMSO-d_6 19

Figure S11. ^{1}H-NMR spectrum of eurocidin E1 in DMSO-d_6 20

Figure S12. ^{13}C-NMR spectrum of eurocidin E1 in DMSO-d_6 21

Figure S13. ^{1}H-^{1}H COSY spectrum of eurocidin E1 in DMSO-d_6 22

Figure S14. HSQC spectrum of eurocidin E1 in DMSO-d_6 23

Figure S15. HMBC spectrum of eurocidin E1 in DMSO-d_6 24

Figure S16. ROESY spectrum of eurocidin E1 in DMSO-d_6 25

Figure S17. ^{1}H-NMR spectrum of eurocidin E2 in DMSO-d_6 26

Figure S18. ^{13}C-NMR spectrum of eurocidin E2 in DMSO-d_6 27

Figure S19. ^{1}H-^{1}H COSY spectrum of eurocidin E2 in DMSO-d_6 28

Figure S21. HMBC spectrum of eurocidin E2 in DMSO-d_6 30

Figure S22. ROESY spectrum of eurocidin E2 in DMSO-d_6 31

Figure S23. TOCSY spectrum of eurocidin E2 in DMSO-d_6 32

Figure S24. Phylogenetic analysis of AT domains from representative T1PKS BGCs 33

Figure S25. SDS-PAGE analysis of wild-type and mutant AT domains from loading modules of polyene T1PKSs 34

Supplementary References 35

Supplementary Tables

Table S1. AT sequences analyzed in this study

Protein	Constructed or synthesized	Sequence
Eurocidin, SalbiC6.1_AT	Constructed	MGSSHHHHHHSSGLVPRGSHMASAEEDSEAGPT GTPEAATGPAAPWILSARSQAALRVQAQRLAERG QARLGLTAQDIAYSLATTRPLHRHRAVISGPGRAEL LSAAEEFGEGKRASGVSVEHSVPGGLAFVFPGGG CQRLGMGREAAEAFPVFAAALHEVCAVVDTMLDR PLTSVMWADPESEEAALIDENRYAQPALFALQVALY RLFESWGVVPDRVAGHSAGETAAVHVAGGLDLKD ACTVVVTRGVLMDSLPPDGVMVAVRVSESEVAPLL AESPGPVAIASVNGPRSLVLAGAEEPLTALTDRLNA AGHKTRRVAVNGAAHSPLMDPVLEEFGAVARTLSY STPAIPVVSTLTGRHLTPEDAHDPDHWVRHLRESV RFTDAVDRLRNDRITGFLELGPQPVLTPLIDECLEA ADPGYGAALVPALNAGESERQALLTAVARMHAHGV PVDWRAVLPDARPV
Candicidin, Cand_AT	Constructed	MGSSHHHHHHSSGLVPRGSHMASAGTERSADAG AAAPRAAVPWLVSAKDADALRGQARRLAAHAAAH PEVSARDLAYSLLTTRALHPRTALLTGGDRDGLVAS ADAFARGEAPGSIVRGPLGPAPGTAFVLTGQGSQR LGMGRGLAAAFPVFDDALREVCALLDPLLERPLTE VMWAAPDSDEAGLLGGTGYAQPALFAFEVALYRLL ESWGIVPDRLVGHSVGEIAAAHVAGVLSLPDACAL VAARGRLMQALPPGGAMAAVRCSEAEILPLLAGRT AGVTVAAVNGPRSVVLSGTEEAVAEVVTEVSAAGH KTRRLMVSHAFHSPLMEPMLAEFRATVAGLSFAAP QVPLVSGVTGRPLTAEEARDPDHWVRHARDTVRF ADAISHLAGEHTEIYVELGPEAALTPMVEECLGEPE SGDGPAVEPVVRGDVDEERAALAAAVRLHALGLDV QWRAVLPEARAVPL
Amphotericin, Amph_AT	Synthesized	MEEAPTTDPAAAVPAGPAHRDVASAADSAARPAAL AGEPADTSAPAAVDAGPADRPVTPAARLAALVPAA DAVAWPVSGASPEALDAQVERLTSFVRDHPGADP LDIGHSLATGRAALRHRAVLVPSGDGVVEIARGEAA PRTTAVLFSGQGSQRLGMGRELAARFPVFAKALDT

		VLAALDPQLERPVRSVMWGEDPAELDRTGWTQPA LFAFEVALYRLAESFGLRPDAVGGHSVGEIAAAHIA GVLSLEDAARLVAARATLMQALPEGGAMSAVEASE DEVLPLLDGDVSLAAVNGPTAVVVSGAEDAVERVS AHFAAQGRRTSRLAVSHAFHSPLMEPMLDAFRDV VAGLTFHEPTLPVMSNLTGELAGAEIATPEYWVRH VRGTVRFADGVTALREHGTDLLVELGPGSVLTALA RTVLGPDTPGAPVDVVPTLRKDQPEERALTAALGR LHVLGATVDWSALYTGTGARRTDLPTYAFQLEHHH HHH
Nystatin, Nys_AT	Synthesized	HHHHHHSSGLVPRGSHMASMTGGQQMGRGSEE APPADVPVTRPGTLRPSTVPWPVSAATPEALDAQL ARLRAHLRTHSDLDPLDVGYSLATGRAALRHRAVL LPPADGTAADAVEHARGAAHQRRTAVLFSGQGSQ RPGMGRELAARFPVFADALDDALRALDRHLDGPV REVMWGTDAALLDRTGWTQPALFAVEVALHRLVAS LGVTPDFVGGHSVGEIAAAHVAGVLSLEDACRLVA ARATLMQALPAGGAMAALEATEDEVAPLLGAHLAL AAVNGPTAVVVAGAEDAVRQLTARFADRGRRTSRL AVSHAFHSPLMEPMLDAFRDVVSRLTFHQPSIPLVS NLTGELAGSEITSAEYWVRHVRDTVRFADGITALAK AGADVLIELGPGGVLSAMARDTLGPDSTTDVVPAL SKGRPEETAFAGALGRLHTLGVPVDWPAFYAGTG ARRVELPTYAF
Eurocidin, Eur_AT	Synthesized	HHHHHHSSGLVPRGSHMAILEEAPAEDDTTGAPEA AAVPAAPWILSARSQAALRAQAQRLAAELGQARLG LTAQDVAYSLATTRALHRHRAVISGPGRAELLSAAA EFGDGKRASGVTIENSTPGGLTMVFPGQGCQRPG MGREAAEAFPVFADALREVCAAMDPLLDRPLISVM WADPDSEEAVLLDEAGYAQPALFAVQVAMYRLFES WGVVPDRLMGHSAGENTAVHVAGGLGLADSCALV ALRGRLMDGLPPGGAMVAVRISESEVKPLLAEASA PLAIASVNGPNSLVLAGAEGPLATLTDRLNAAGHKT RRIAVNGAAHSPLMDPMLEELGTIARGLSYATPAIP VVSTVTGRLMTPEDAHDPGHWVRHAREAVRFTDA VDRLRDEQITGFLELSPQPVLTPMIDDCLEAADPGY

		GAALVPTLKAGESERQALLTAVARMHAHGVPVDWS AVLPDARPVALPTYPFQ
Rimocidin, Rim_AT	Synthesized	HHHHHHSSGLVPRGSHMASMTGGQQMGRGSEE APDGTQSAPGSEPADDTAVPWVLSARSRTALREQ ARRLAEHVTAHPGLRTQDIAHALATTRTRHRHRAV VSGSDRDRMLSATAAFGRGERAADVTPLDSAPGG LAFVFSGQGGQHPGMGRGAAEAFPVFGEALREV CDTLDPLLARPLTSVMWADADSEEATLLHNAEFSQ PSLFALQVALYRLYESWGMAPDRLAGHSAGEIAAA HVTGILTLQDACALVASRGRLISSLPVGGATVAVRIS EDEVRGWLAEETTGSVSIAAVNGPHSLVLSGAEAP LIALTDRLRDAGHKTHRIPMRVAAHSPLMDPILGEF RAVVRTLAYGTPTIPLVSTVTGRPLTDEEARDPEHW VRHVRQPVRFKDAIGRLREERVTGFLELGAEPLLT PMIDECLEAAGPQHGTAVVPSLSSGVPDRQILLSAA ARVHTHGAPVDWDAVLPGARPVDLPTYAFQRRRF WLA
Natamycin, Nat_AT	Synthesized	HHHHHHSSGLVPRGSHMASMTGGQQMGRGSVIL EEAPGEAAAGARAEVPEEARCASSPARLPEPSGD AAAPWVLSARSRAALRAQALRLADQVAADPGLRA QDVAHALATSRTLHRHRAVVSGSDRAQMLAAAKR FGLGERTAGVTPDDSAPGLLAFVFSGQGSQRSGM GRAAAEAFPVFGRALGEVCAALDPLLTRPLTSVMW AAPGSEEAARLDDTTYTQPALFAVQVALYRLFESW GVVPDQLVGHSVGEISAAHVAGVLGLRDACTLVAA RSRLMGALPPGGAMVAVRITEPEVTPWLAELTDEV SIAAVNGPHSLVLAGAEAPLVALTDRLAAAGHKTRR LMVSTAPHSPLMDPMLEEFRAVVRTLSYAAPAVPLV STVTGRPLTGEEARDPDHWVRHVRQSVRFKDAIG RLRDERVTGFLELGAEPALTPMIDECLESADGQPG TALVPSLRAGVPERDALLTAVARVHAQGVPVDWDA VLPGARPVAL
Tetramycin, Tet_AT	Synthesized	HHHHHHSSGLVPRGSHMASMTGGQQMGRGSEE PPAAAAQEPTGEVPAGAPAGAPEPTGEPAVPWVV SARSPAALRAQARRLAEHVAADPALRAQDVAHALA TTRAVHRHRAVVSGADRDQLLAAALRFGRGEGSS GVTPHDAAPGDLAFVFSGQGSQRNGMGRTAAEAF

		PVFGRALREVCAALDPLLERPLTSVMWAAPDSDEA ALLDDTTYTQPALFAVQVALYRLFESWGVAPDHLVG HSVGEISAAHVAGVLGLRDACTLVAARSRLMGALP PGGAMVAVRITEDEVLPWLAEVADSVAVAAVNGPH SLVLSGAEEPLVALTDRLAAAGHKTRRLTVSTAPHS PLMEPMLAAFREVVGALSFSAPAVPLVSTVTGRPLT EAEARDPEHWVRHVRQSVRFRDAIDRLREARVTG FLELGAEPALTPMIDECLESDAAQPGAAVLPSLRGG ASERQALLTAVARLHLHGVPVDWDAVLPGVRGVPL PTYAFQRRRFWLA

Table S2. Plasmids, primers, and sequences used in this study

Plasmid	Primer name	Primer sequence
pET28a-Nhis-salbi_AT	salbi_AT-F	CGGCAGCCATATGGCTAGCGCCGAGGAGGACTCCGAAG
	salbi_AT-R	AGTGCGGCCGCAAGCTTGTCAAACCGGCCGGGCGTC
pET28a-Nhis-cand_AT	candi_AT-F	CGGCAGCCATATGGCTAGCGCCGGCACGGAGCGGAG
	candi_AT-R	AGTGCGGCCGCAAGCTTGTCACAGCGGCACCGCGCG
pET28a-Nhis-salbi_ATmtNGAA-SHAF	NGAA-SHAF-F1	CGGTGAGCCATGCGTTTCACTCCCCGCTCATGGATC
	NGAA-SHAF-R1	TACTGAGAGTGCACCATATATGC
	NGAA-SHAF-F2	TATATGGTGCACTCTCAGTACAATC
	NGAA-SHAF-R2	GGAGTGAAACGCATGGCTCACCGCGACCCGCC
pET28a-Nhis-cand_ATmtHAF-GAA	HAF-GAA-F1	GGTCAGCGGAGCTGCTCACTCGCCGCTGATGGAGC
	HAF-GAA-R1	CTTAACTATGCGGCATCAGAGCAGATTGT
	HAF-GAA-F2	TCTGATGCCGCATAGTTAAGCCAGTA
	HAF-GAA-R2	GCGGCGAGTGAGCAGCTCCGCTGACCATGAGCCGAC
pKC1139-fscA	L-F	CGGCTCGTATGTTGTGTGGACGTGATGGGCGACGCCTCA
	L-R	ACGGGGCGCCCACTCCCGGGATGTCGAGC
	R-F	CCCGGGAGTGGGCGCCCCGTGACTCTCT
	R-R	TAGCTGCGCCGATGGTTTCTCGAAGAGGATGACCACCGGC
pSETW101-salbi_LD	LD1-F	CGTGCAGGACTGGGGGAGTTATGGTGTCCGTCCACACAGATGACT
	LD1-R	CGGTGCAGAGCATGCCTTCCAG
	LD2-F	TGGAAGGCATGCTCTGCACCG

	LD2-R	GCTCGTATGTTGTGTGGAATTCACCCGACCTCGTCCATCAGG
pSETW101-salbi_NRPS_PKS+cand_DD	NRPS_PKS-F	CGTGCAGGACTGGGGGAGTTATGGTGTCCGTCCACACAGATGACT
	NRPS_PKS-R	AACCGGACGTCGCCAGCCAGAACCGCCG
	DD-F	CTGGCTGGCGACGTCCGGTTCCGTGGTCG
	DD-R	CTCGTATGTTGTGTGGAATCGGGCGGAGAGAGTCACGG

Supplementary Figures

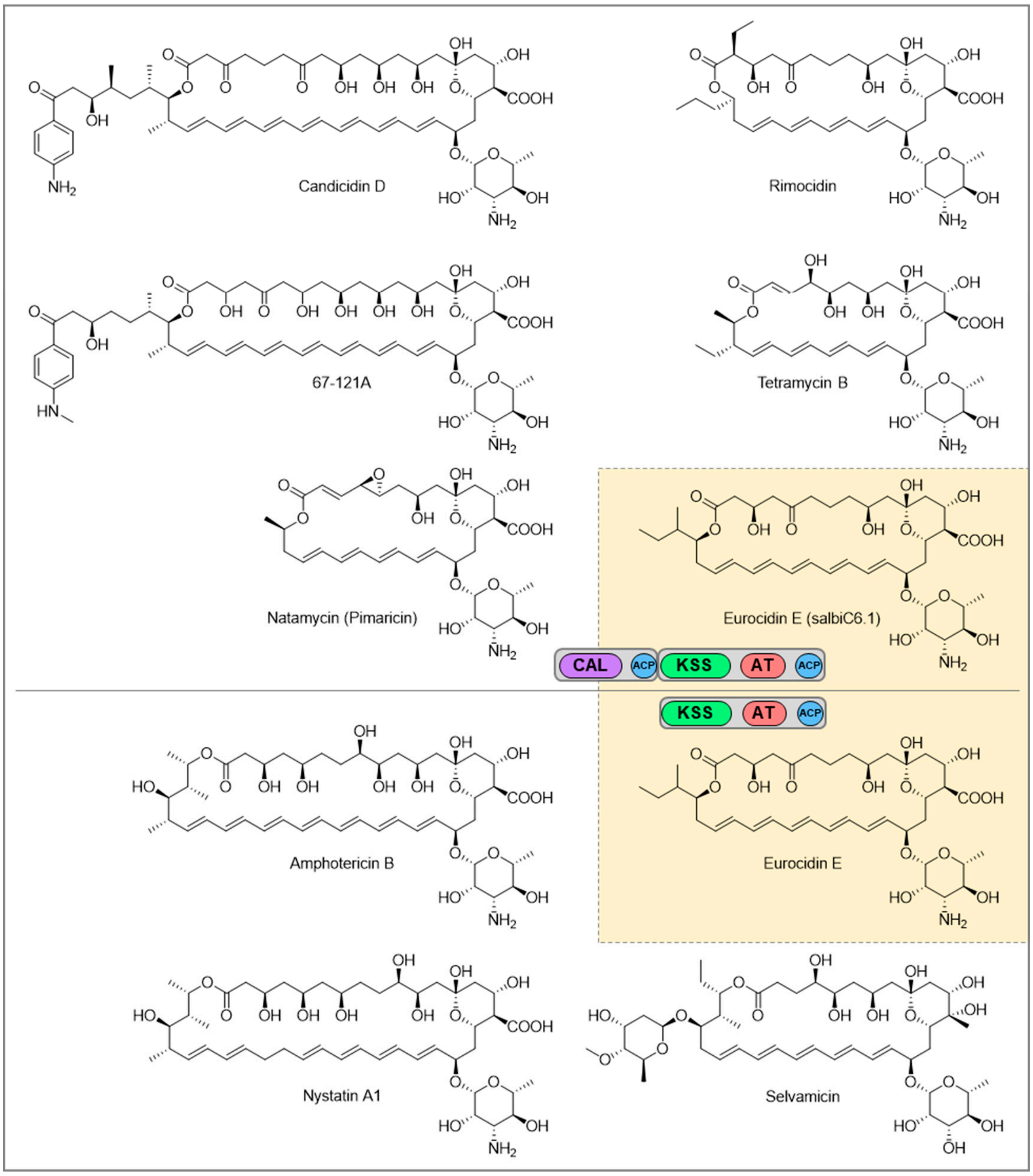

Figure S1. Representative polyene macrolides and corresponding biosynthetic loading module architectures.

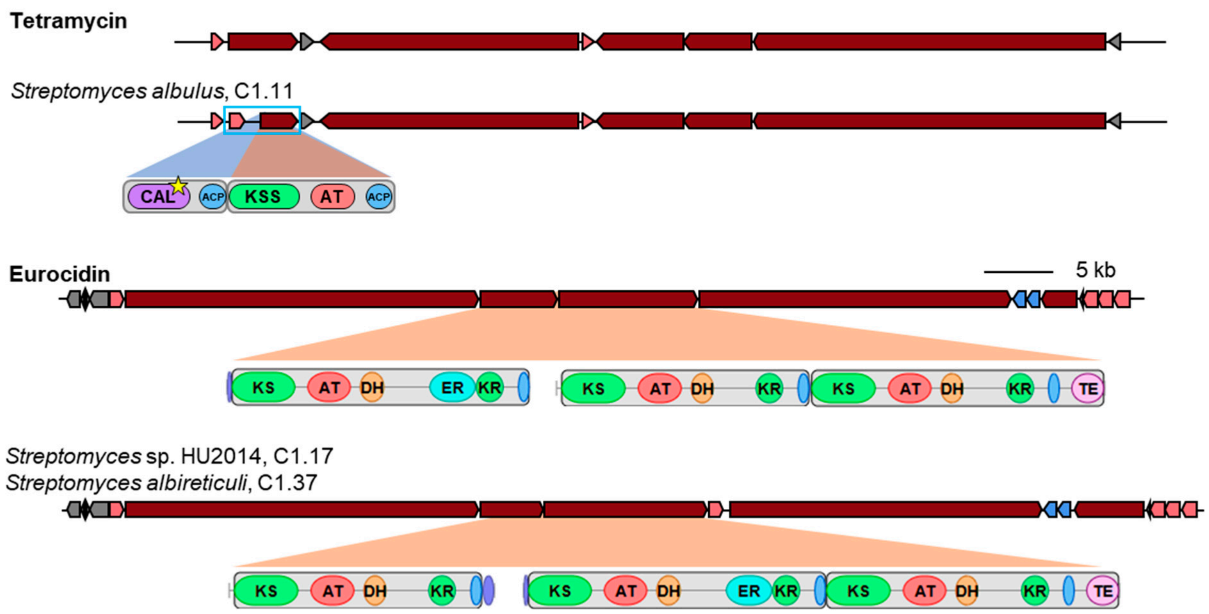

Figure S2. Tetramycin- and eurocidin-like biosynthetic gene clusters identified from antiSMASH database. For tetramycin, a sequencing error (yellow star) led to misannotation of the CAL domain. For the eurocidin search, identical gene clusters were discovered from two different strains, but their module composition is distinct from the reference.

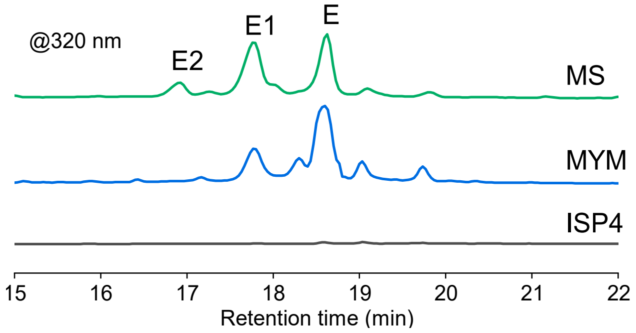

Figure S3. Metabolic profiles of *Streptomyces albireticuli* in three media.

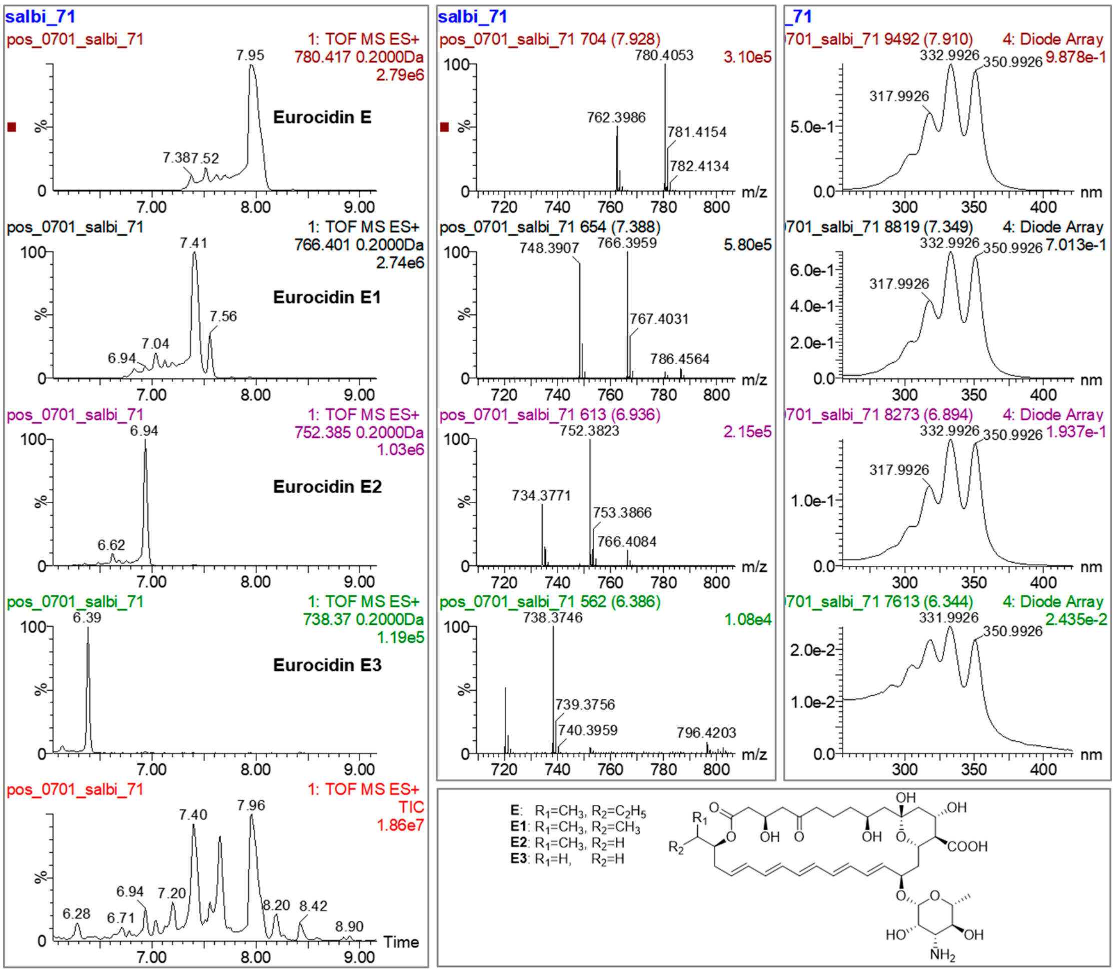

Figure S4. LC-HRMS data of fermentation products from *S. albireticuli*. Columns from left to right are extracted ion spectra (EIC), mass spectra, and UV-vis spectra.

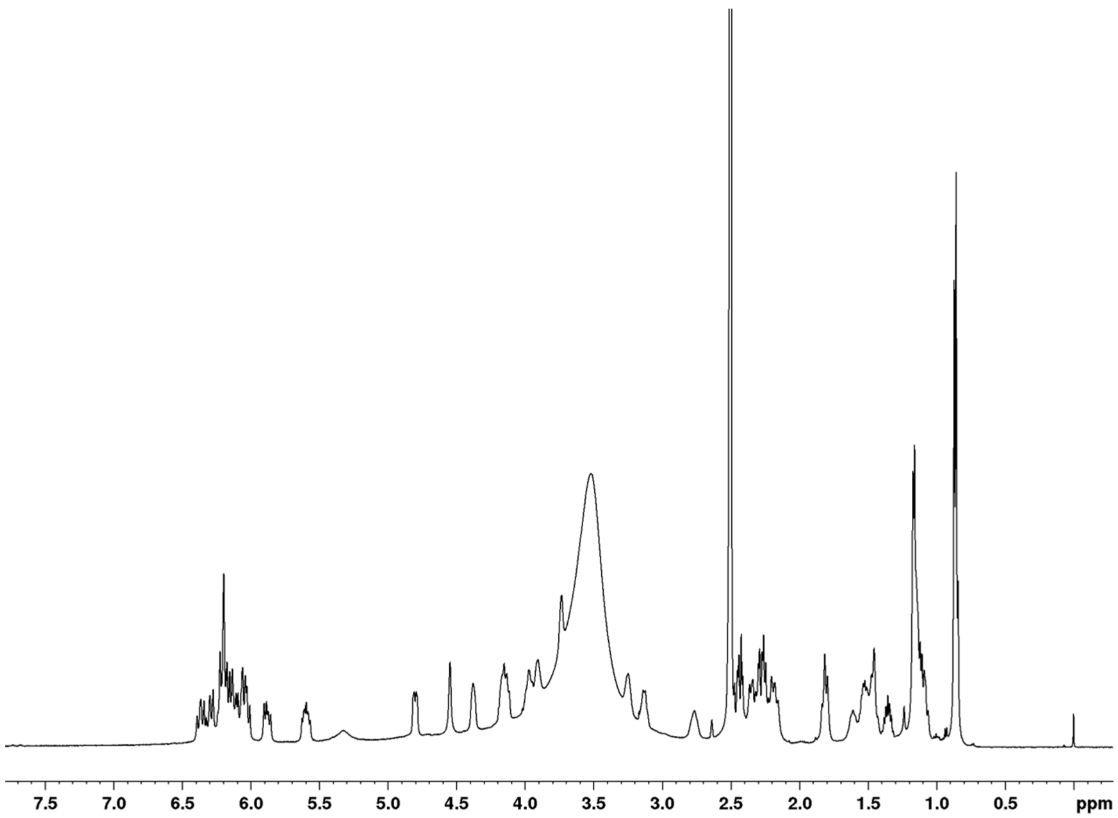

Figure S5. ^{1}H-NMR spectrum of eurocidin E in DMSO-*d6*.

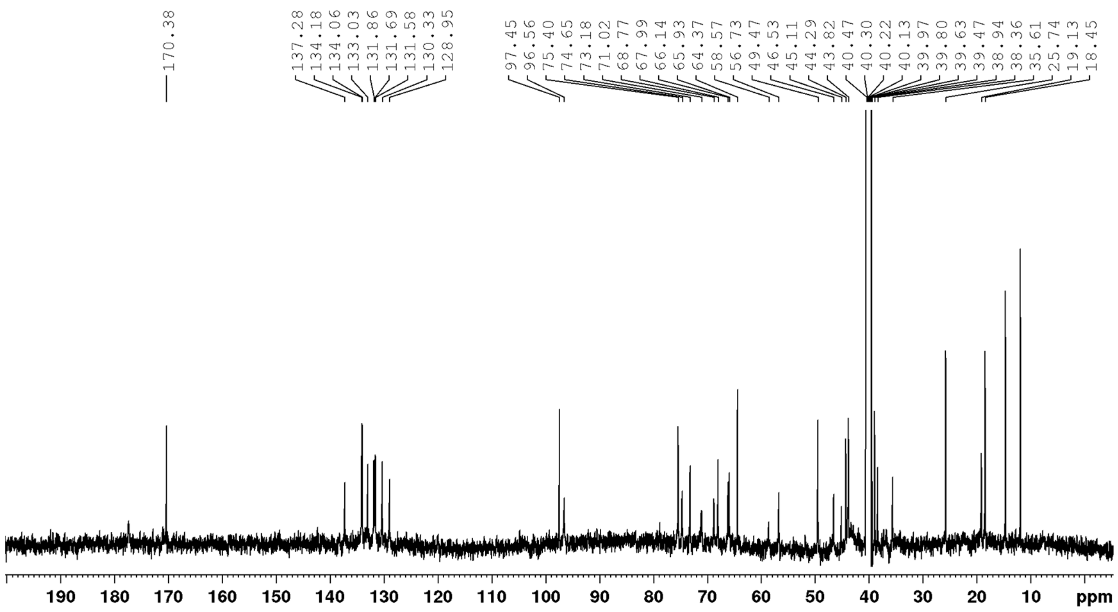

Figure S6. ^{13}**C-NMR Spectrum of eurocidin E in DMSO-*****d*****$_6$.**

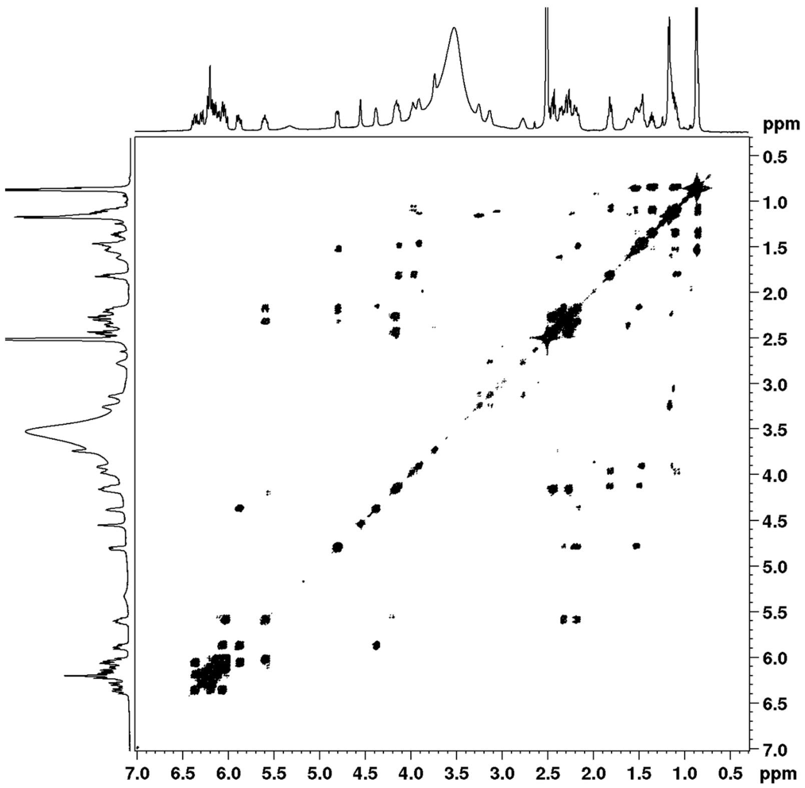

Figure S7. ^1H-^1H **COSY spectrum of eurocidin E in DMSO-*d*$_6$.**

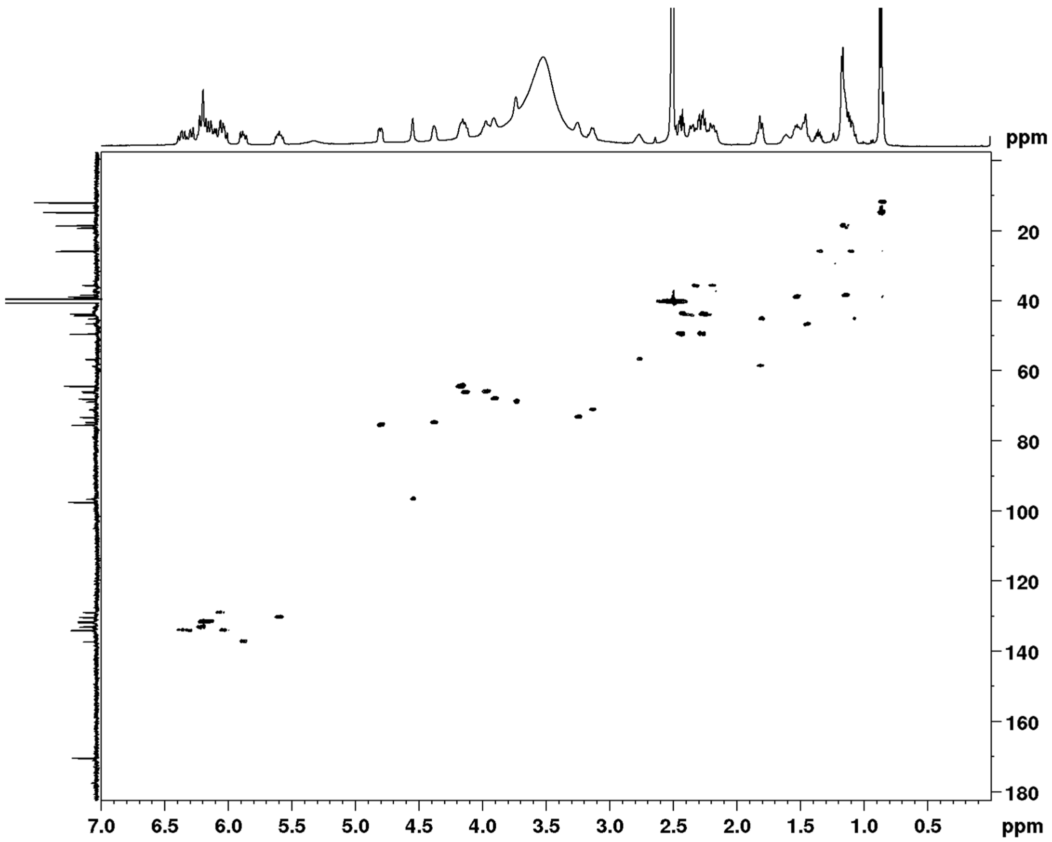

Figure S8. HSQC spectrum of eurocidin E in DMSO-d_6.

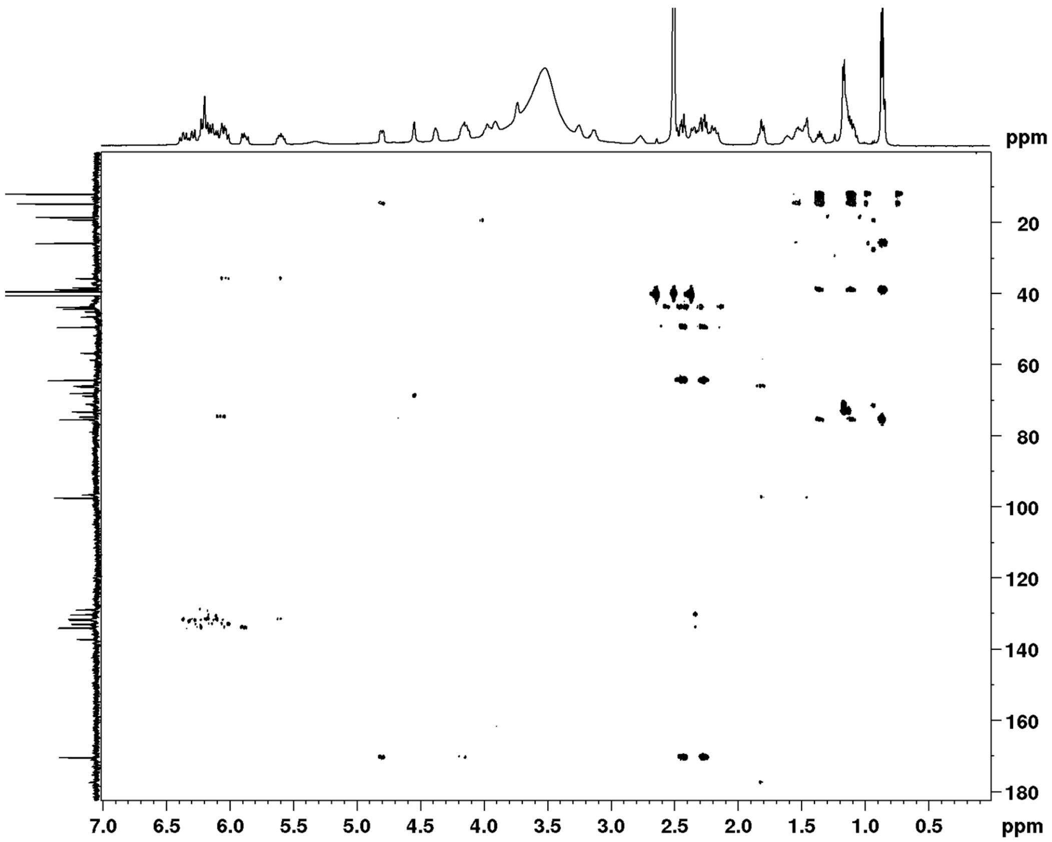

Figure S9. HMBC spectrum of eurocidin E in DMSO-d_6.

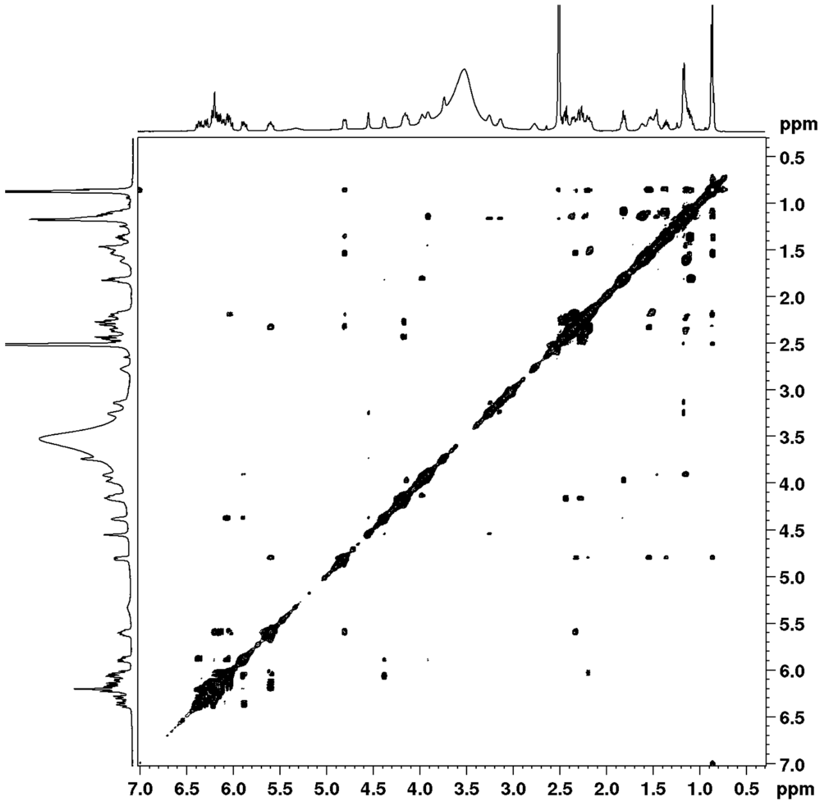

Figure S10. ROESY spectrum of eurocidin E in DMSO-d_6.

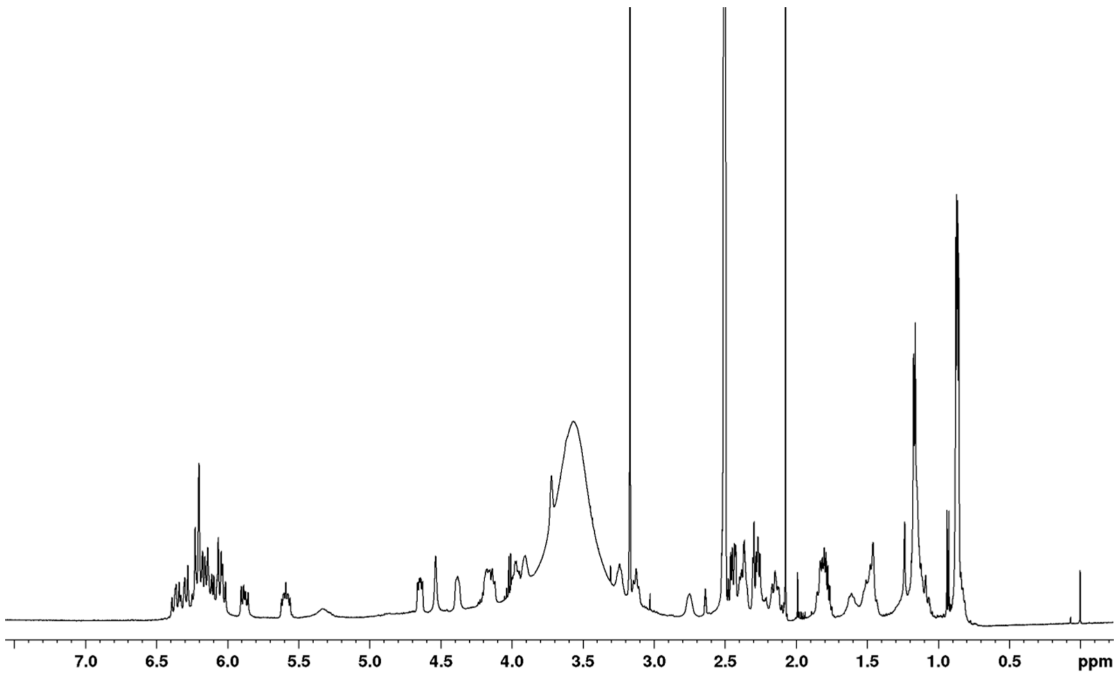

Figure S11. ^{1}H-NMR spectrum of eurocidin E1 in DMSO-d_6.

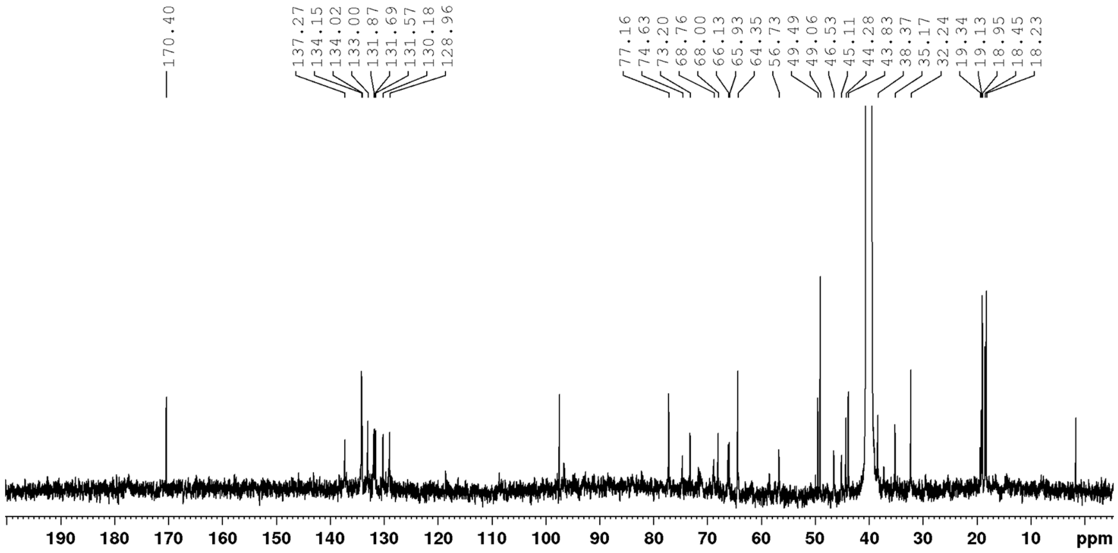

Figure S12. **^{13}C-NMR spectrum of eurocidin E1 in DMSO-*d*$_6$.**

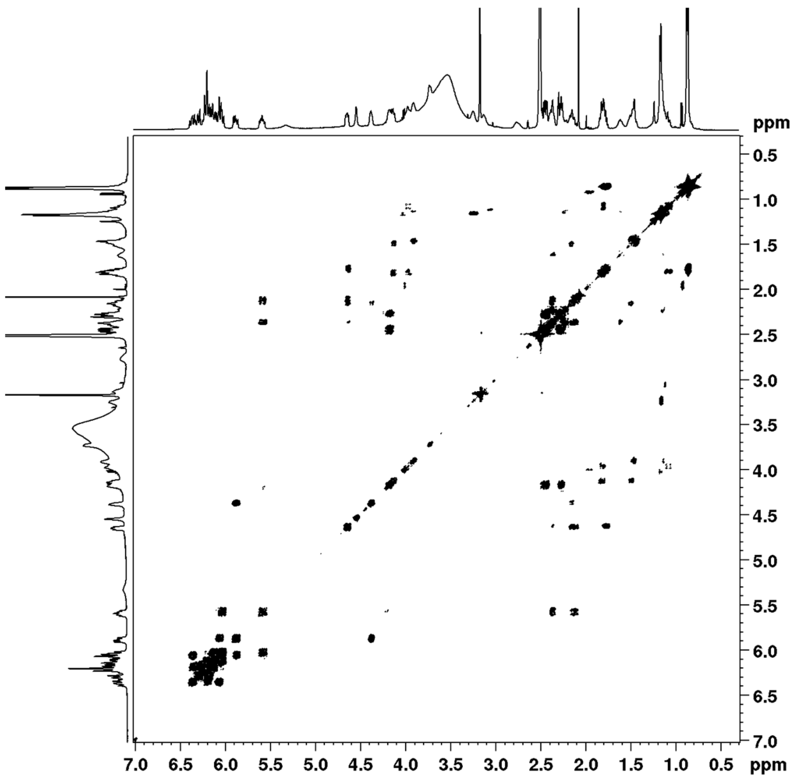

Figure S13. ^{1}H-^{1}H COSY spectrum of eurocidin E1 in DMSO-*d*$_6$.

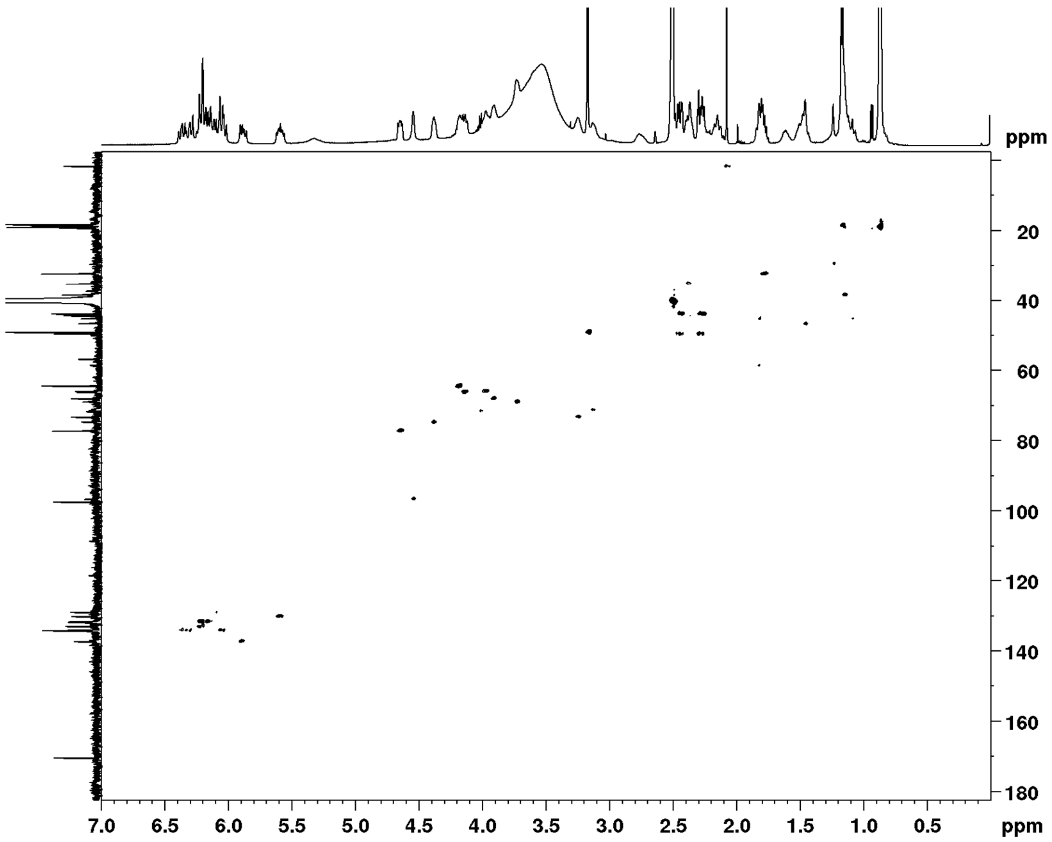

Figure S14. HSQC spectrum of eurocidin E1 in DMSO-d_6.

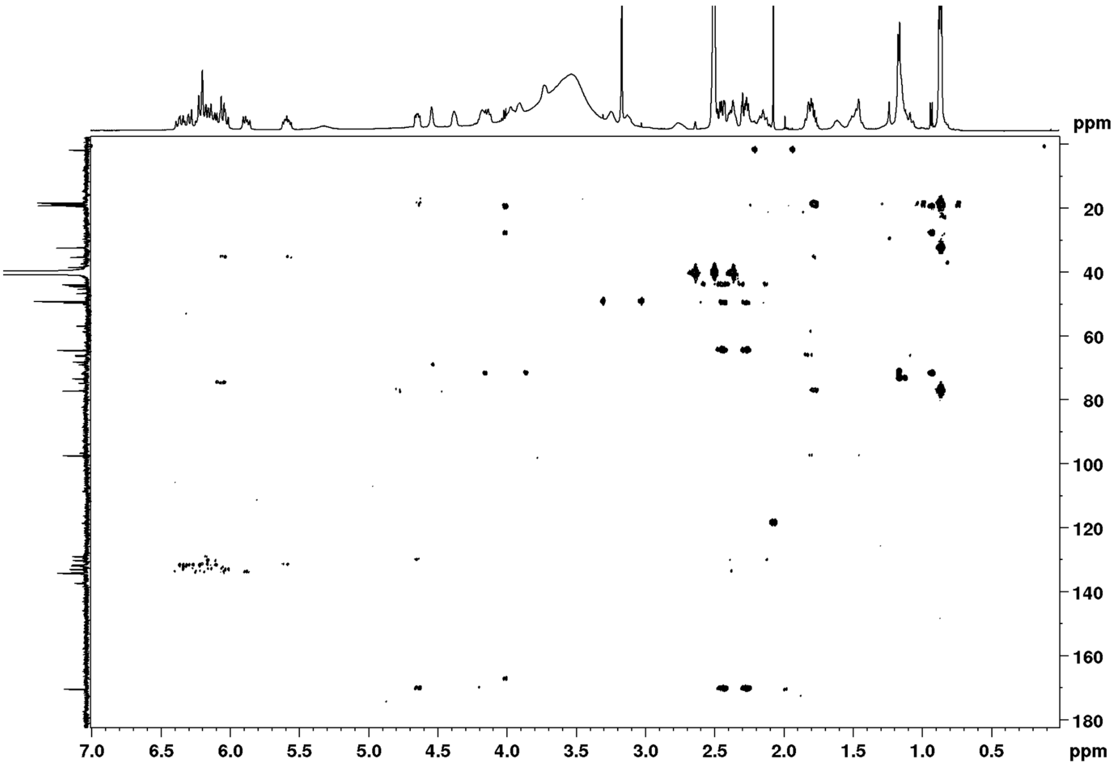

Figure S15. HMBC spectrum of eurocidin E1 in DMSO-d_6.

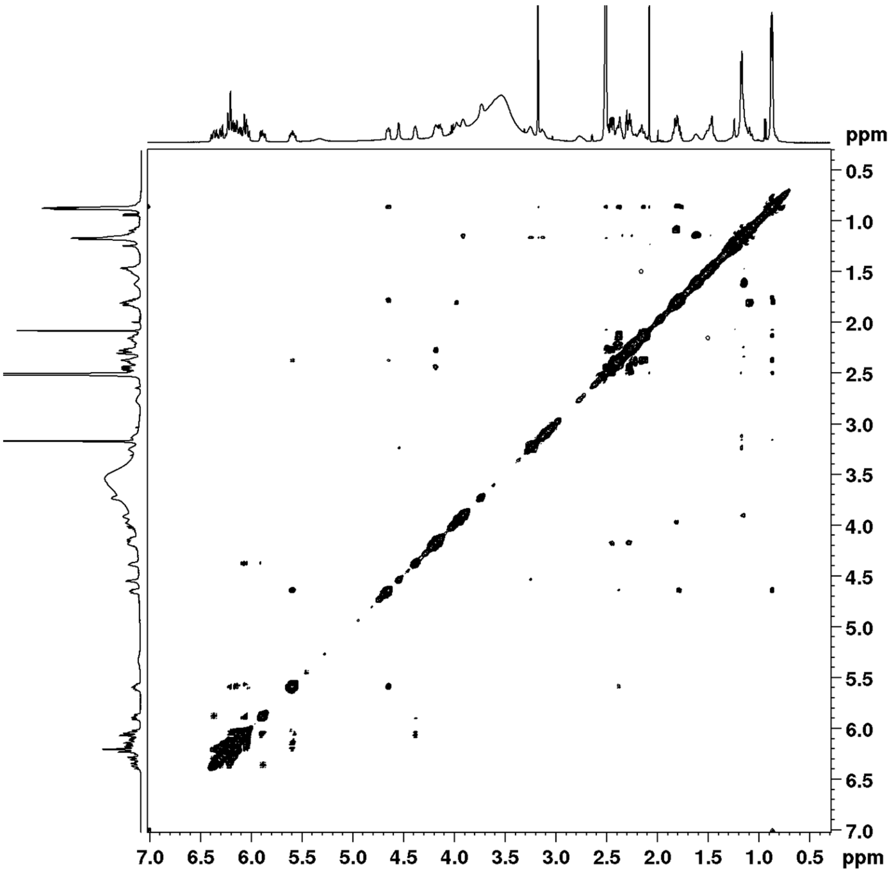

Figure S16. ROESY spectrum of eurocidin E1 in DMSO-*d*6.

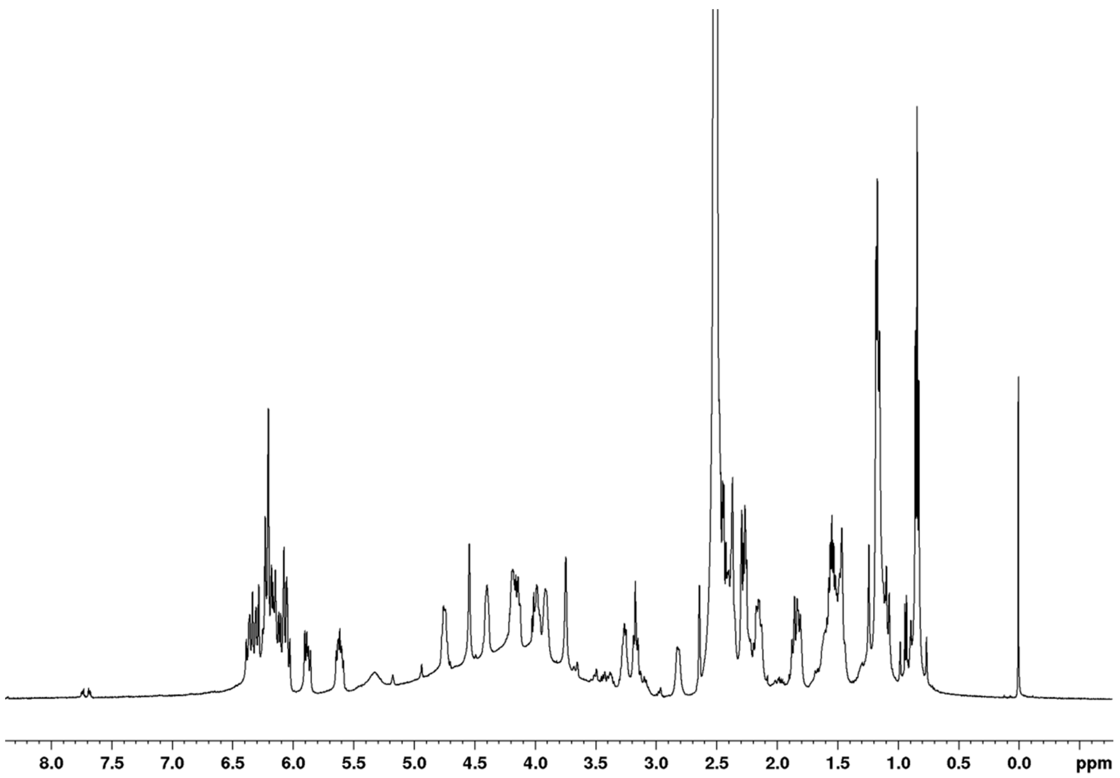

Figure S17. ^{1}H-NMR spectrum of eurocidin E2 in DMSO-d_6.

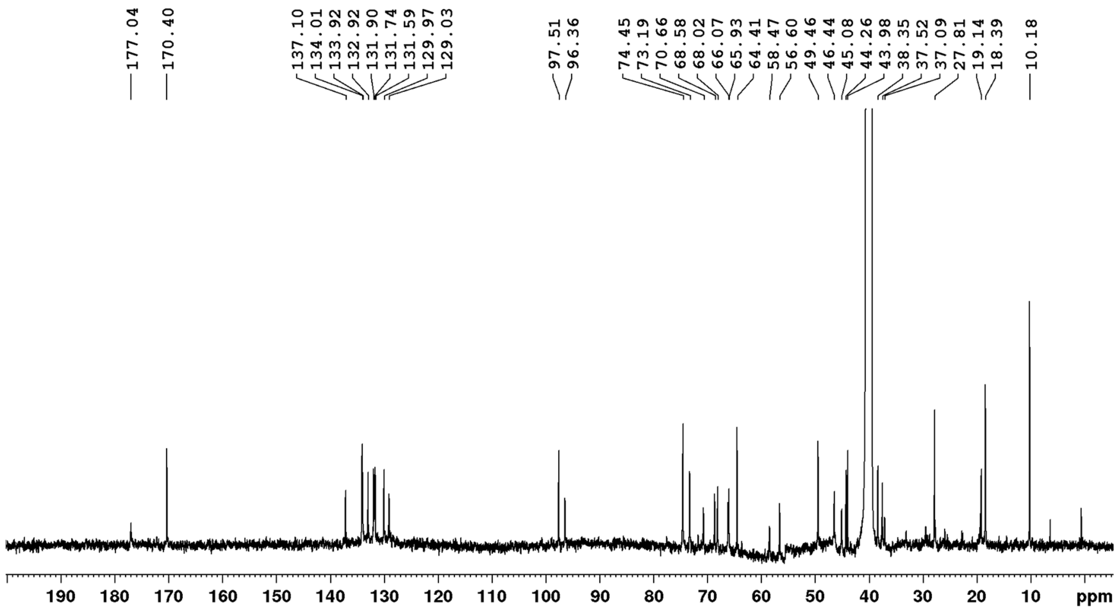

Figure S18. **^{13}C-NMR spectrum of eurocidin E2 in DMSO-d_6.**

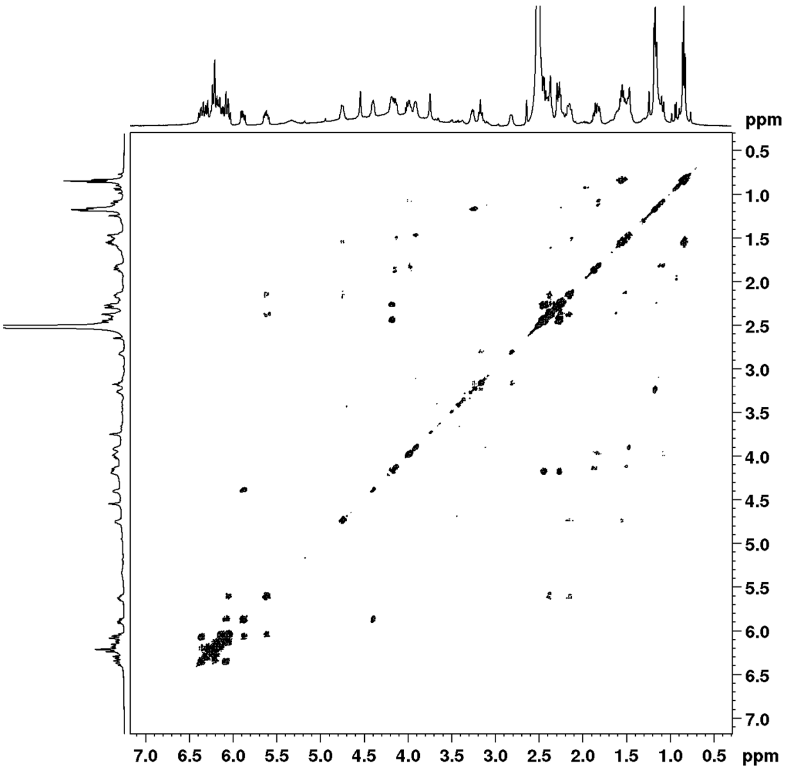

Figure S19. ^{1}H-^{1}H COSY spectrum of eurocidin E2 in DMSO-d_6.

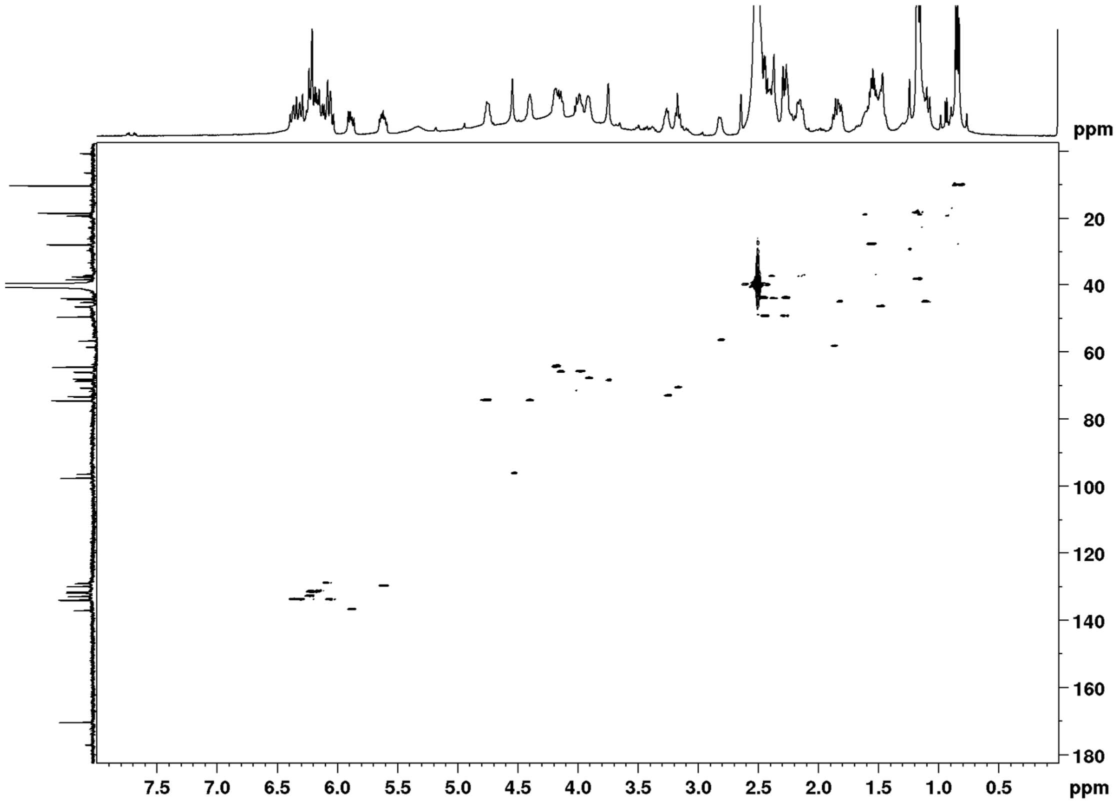

Figure S20. HSQC spectrum of eurocidin E2 in DMSO-*d*$_6$.

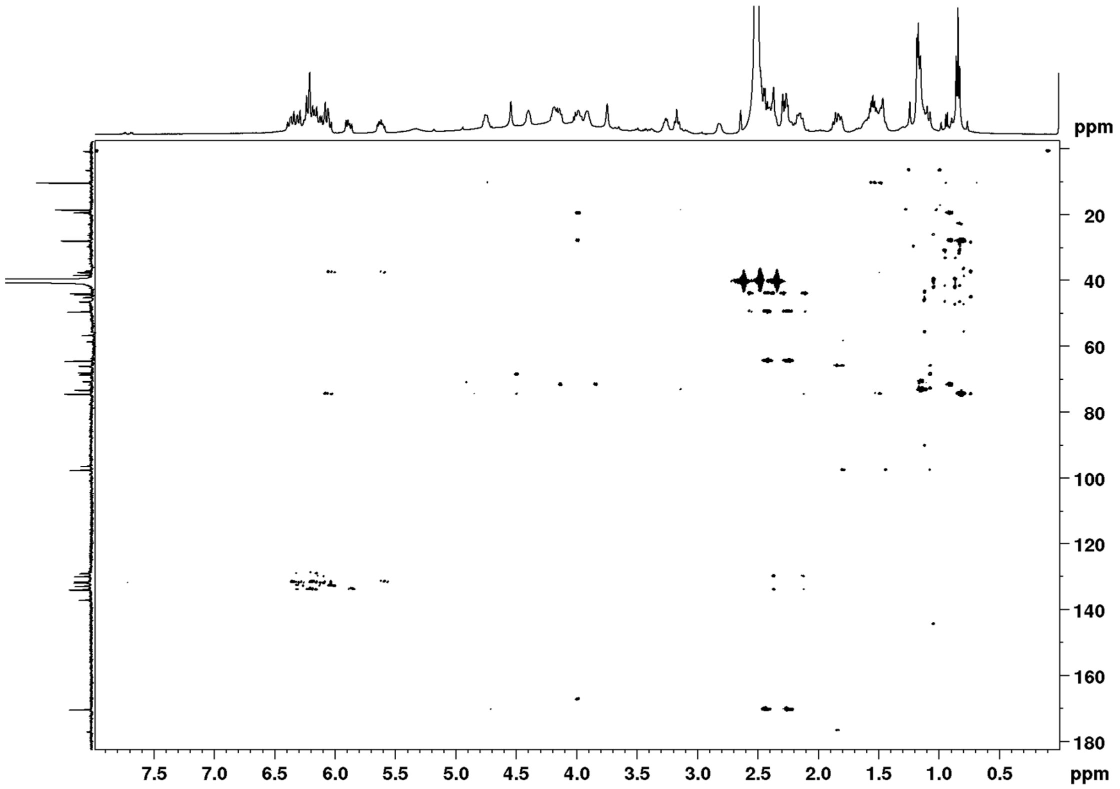

Figure S21. HMBC spectrum of eurocidin E2 in DMSO-d_6.

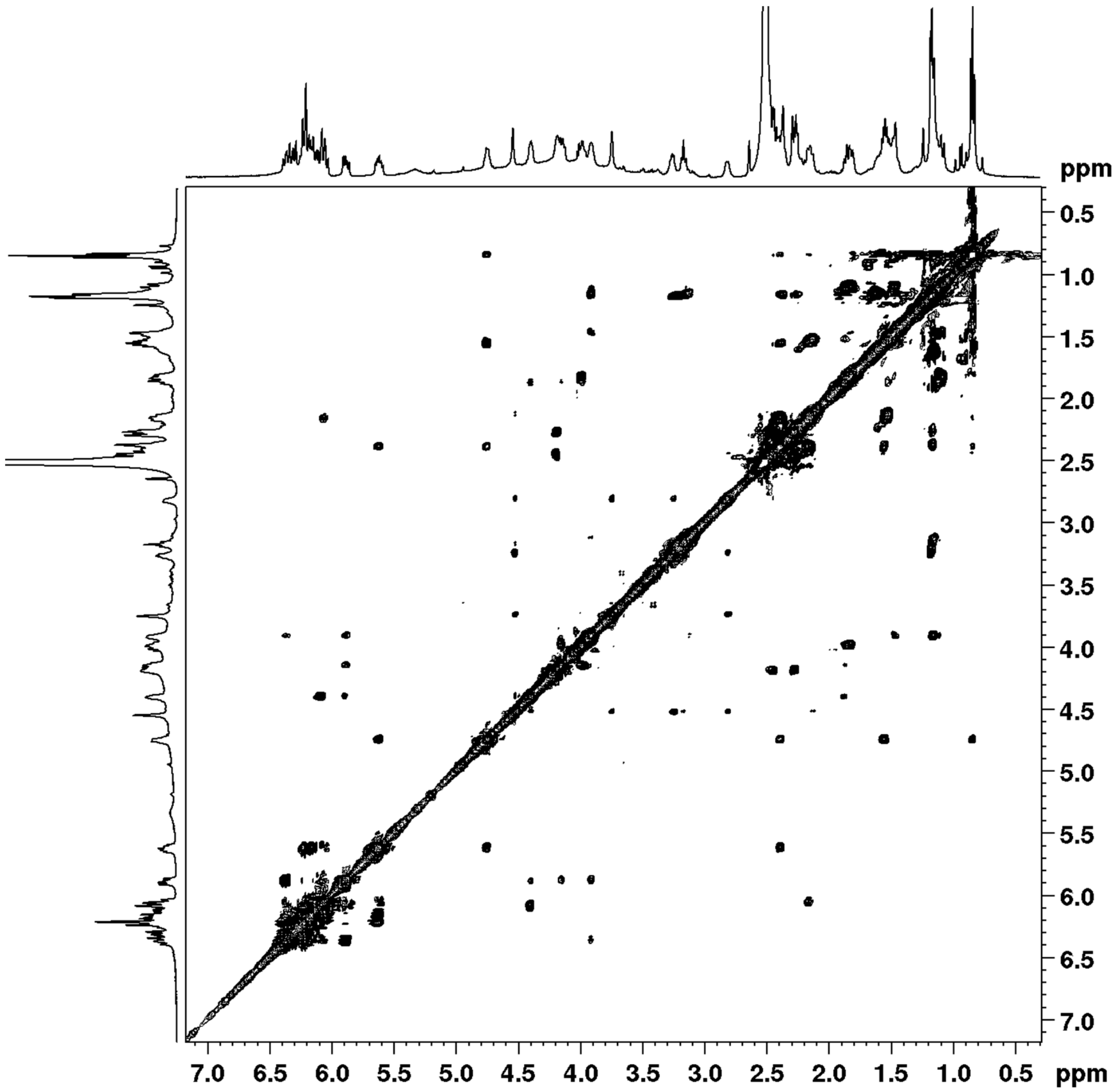

Figure S22. ROESY spectrum of eurocidin E2 in DMSO-d_6.

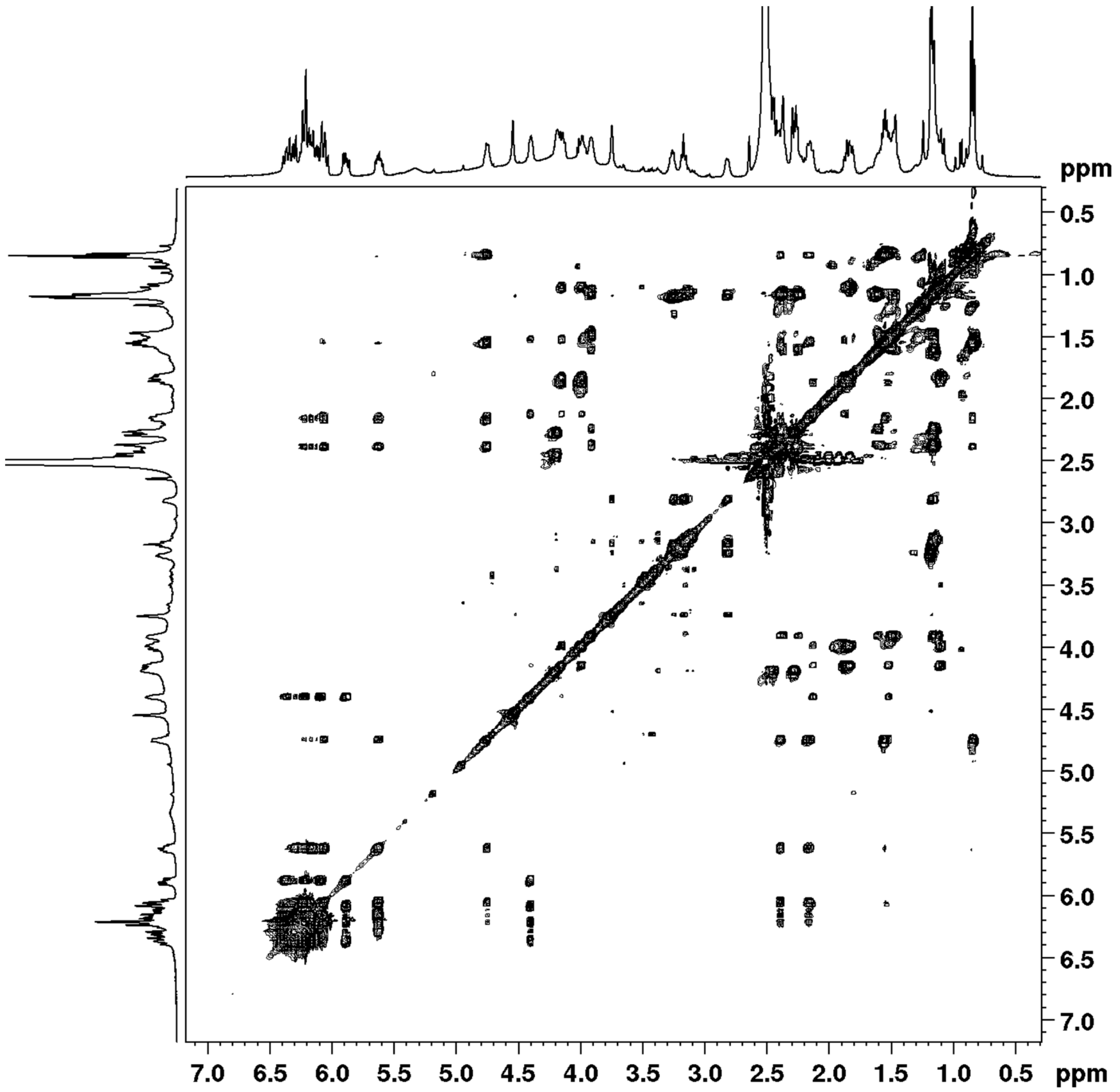

Figure S23. TOCSY spectrum of eurocidin E2 in DMSO-*d*$_6$.

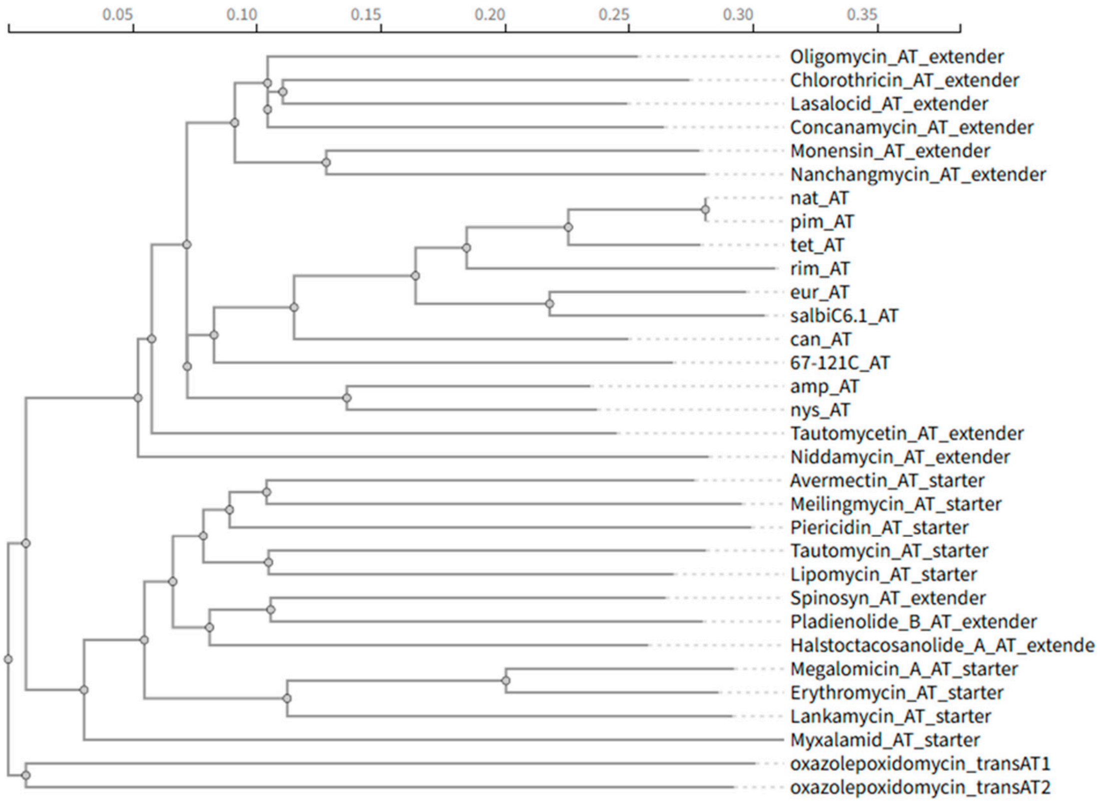

Figure S24. Phylogenetic analysis of AT domains from representative T1PKS BGCs. Sequences were extracted according to antiSMASH[1] annotation. The phylogenetic tree was performed by MUSCLE[2].

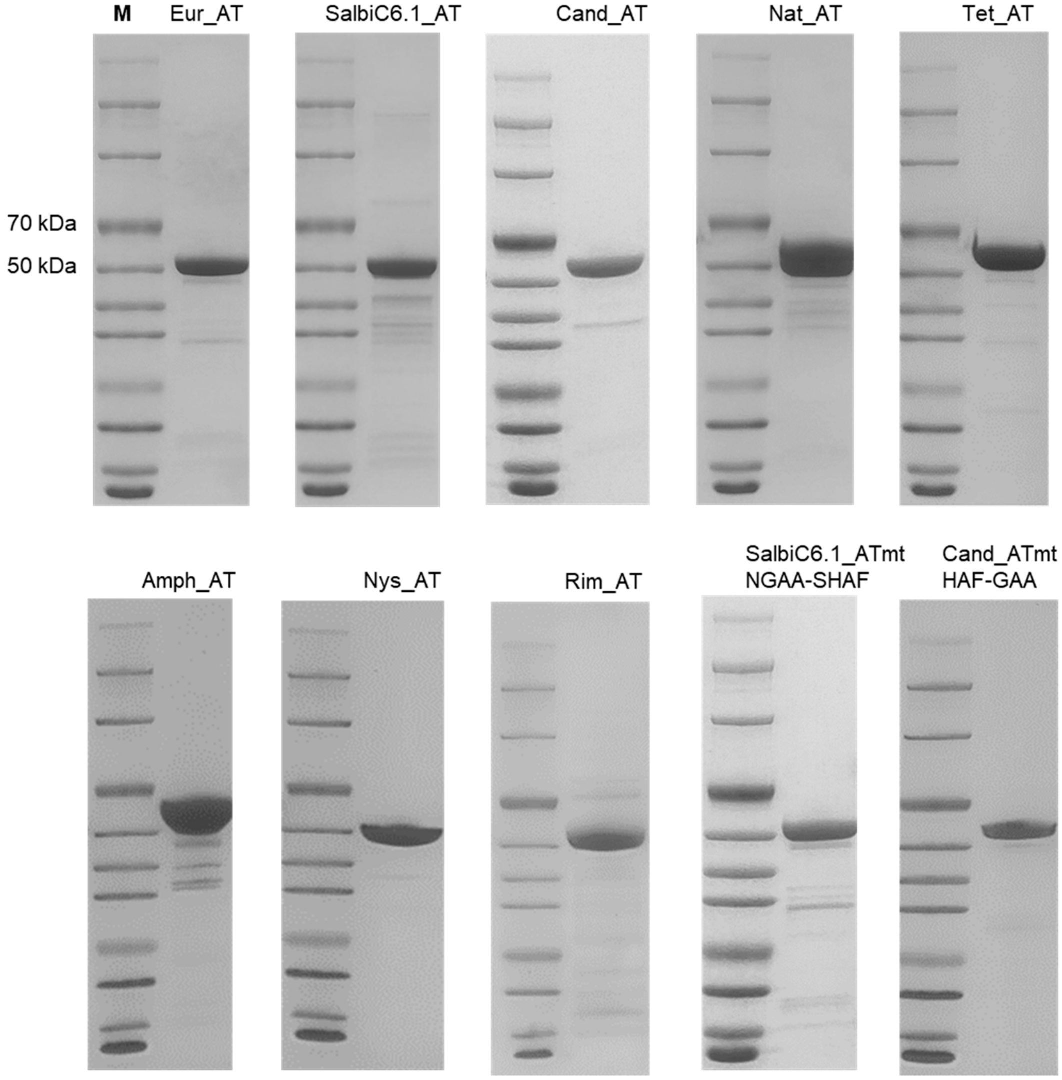

Figure S25. SDS-PAGE analysis of wild-type and mutant AT domains from loading modules of polyene T1PKSs.

Supplementary References

1. Blin, K.; Shaw, S.; Augustijn, H.E.; Reitz, Z.L.; Biermann, F.; Alanjary, M.; Fetter, A.; Terlouw, B.R.; Metcalf, W.W.; Helfrich, E.J.N.; et al. antiSMASH 7.0: New and Improved Predictions for Detection, Regulation, Chemical Structures and Visualisation. *Nucleic Acids Res.* **2023**, *51*, W46–W50,.
2. Madeira, F.; Madhusoodanan, N.; Lee, J.; Eusebi, A.; Niewielska, A.; Tivey, A.R.N.; Lopez, R.; Butcher, S. The EMBL-EBI Job Dispatcher Sequence Analysis Tools Framework in 2024. *Nucleic Acids Res.* **2024**, *52*, W521–W525,.
